# K_V_1.8 (*Kcna10*) potassium channels enhance fast, linear signaling in vestibular hair cells and facilitate vestibulomotor reflexes and balance

**DOI:** 10.1101/2025.01.28.634388

**Published:** 2025-01-28

**Authors:** Hannah R. Martin, Brandie Morris Verdone, Omar López-Ramírez, Merrill Green, Dana Silvian, Emily Scott, Kathleen E. Cullen, Ruth Anne Eatock

**Affiliations:** 1 University of Chicago, Department of Neurobiology; 2 Johns Hopkins University, Department of Biomedical Engineering

**Keywords:** potassium channels, hair cells, vestibular, receptor potential, vestibulo-ocular reflex, balance

## Abstract

Vestibular hair cells (HCs) faithfully and rapidly detect head motions and gravity, driving motor reflexes that stabilize balance and gaze during locomotion. With the transition from water to land, the amniote vestibular inner ear added type I HCs, which differ from amniote type II HCs and anamniote HCs by their large calyx afferent synapse, non-quantal afferent transmission, and a large, low-voltage-activated K^+^ conductance (g_K,L_). We recently showed that both g_K,L_ and the major type II K^+^ conductances (A-type and delayed rectifier) require K_V_1.8 (*Kcna10*) subunits. Here we compared K_V_1.8-null (*Kcna10*^−/−^) and control animals to see how K_V_1.8 affects function as measured by receptor potentials and nonquantal postsynaptic potentials evoked by direct hair bundle motions, and by vestibulomotor behaviors. Recordings were taken from extrastriolar zones of the utricle. In both HC types, K_V_1.8 affected receptor potentials by reducing response time and gain, increasing dampening, and expanding the frequency bandwidth toward high frequencies. Effects are most prominent in type I HCs: lowpass corner frequencies of receptor potentials in *Kcna10*^−/−^ HCs of both types were ~20 Hz, *vs*. ~400 Hz in control type I and ~70 Hz in control type II. We recorded nonquantal postsynaptic potentials from extrastriolar calyces, and found that the synaptic transfer function had lower gain and greater phase lag in *Kcna10*^−/−^ mice. In behavioral tests, *Kcna10*^−/−^ mice had vestibular-ocular reflexes with different response dynamics at low frequencies, impaired performance on a narrow balance beam, abnormal body posture and abnormal head motions in water and on land, and also rarely assumed bipedal stances. These vestibulomotor deficits in *Kcna10*^−/−^ mice likely reflect the changes noted in HCs, where K_V_1.8 expression is concentrated; that is, slower signaling of high-frequency head motions by *Kcna10*^−/−^ HCs fails to fully stabilize body and head position during locomotion. Thus, g_K,L_ (K_V_1.8) contributes to fast signal transmission in the amniote vestibular inner ear and supports improved performance on challenging vestibulomotor tasks.

## Graphical Abstract

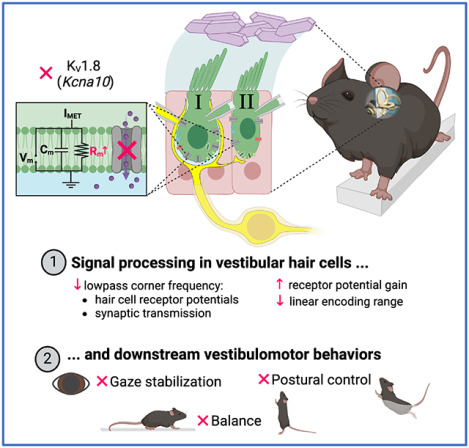

## Introduction

The vestibular sense of self-motion and gravity is necessary for effective motor coordination and navigation. Reflecting this, the vestibular inner ear underwent multiple changes as amphibians transitioned from an aqueous to terrestrial environment to become stem reptiles^1,2^. Modern amniote descendants (reptiles, birds, mammals) alone have specialized type I vestibular hair cells (HCs), in addition to more conventional type II HCs. Similarities in type I HCs across amniote classes suggest that they evolved in stem reptiles as an adaptive change that benefits terrestrial locomotion by increasing the speed and linearity of signal transmission^3–5^.

Type I HCs are enveloped by a calyceal afferent terminal^6,7^ and express g_K,L_: a large, low-voltage-activated K^+^ conductance^8–10^. Unlike outwardly rectifying K^+^ conductances in other HCs, including type II HCs, which tend to activate positive to resting potential (V_rest_), g_K,L_ is substantially open at rest, lowering input resistance and consequently reducing the gain and membrane charging time of the receptor potential^11,12^. At the type I-calyx synapse, g_K,L_ also participates in non-quantal transmission (NQT), an unusual coupling that reduces synaptic delay relative to quantal glutamate release alone. During a HC receptor potential, K^+^ efflux from basolateral HC K channels, predominantly g_K,L_, raises [K^+^] in the cleft^5^, which depolarizes the afferent by via two mechanisms: a fast ephaptic potential in the cleft^13^, and a slower elevation of the K^+^ equilibrium potential (E_K_)^14–16^. NQT may also benefit from modulation by proton and glutamate accumulation^17,18^, but NQT-mediated depolarization can drive afferent activity independent of glutamate-based transmission^19–21^. The impacts of NQT on vestibular function have been suggested based on its extraordinary properties^22^, but have not been directly tested. In contrast, type II HCs transmit by quantal (vesicular) release of glutamate to small bouton terminals and have more conventional delayed rectifier and fast-inactivating A-type (g_A_) K^+^ conductances.

We recently found that g_K,L_, g_A_, and part of the delayed rectifier in type II hair cells all require K_V_1.8 subunits (gene name *Kcna10*)^23^. *Kcna10*^−/−^ mice lack a vestibular-evoked potential (VsEP), which represents the synchronized activity of saccule striolar afferents responding to fast linear head motions^24^. Most striolar (central) or extrastriolar (peripheral) zone afferents integrate signals from both type I and II HCs^25^ and then project to either the cerebellum or vestibular nucleus (VN)^26,27^. Peripheral VN-projecting afferents drive the vestibular-ocular reflex (VOR)^28^, a 5–6 ms reflex that maintains a stable visual field during head motions^29,30^. The VOR and other postural and balance reflexes rely on a precise, context-independent^31^ readout of head motion from vestibular afferents.

Given the importance of signal conditioning by ion channels like K_V_1.8, we asked how knocking out K_V_1.8 would affect each level of vestibular function, from receptor potentials and synaptic transmission to gaze stabilization, balance, and postural control. To understand the contributions of K_V_1.8 to signal processing and vestibulomotor behavior, we compared receptor potentials, NQT, the VOR, and other vestibulomotor behaviors between *Kcna10*^−/−^ mice and littermate controls. We conducted patch clamp electrophysiology while displacing hair bundles in the extrastriola of the mouse utricle.

Together, we identify an essential role for the K_V_1.8 subunit in conditioning receptor potentials to enable temporally faithful vestibular output, precise compensation for head and body movements, and postural refinement, supporting the adaptive appearance of type I HCs during the transition to life on land.

## Results

To explore the role of K_V_1.8 in transmission of head-motion signals, we began by recording from type I and II HCs and calyces from the medial and lateral extrastriolar zones (MES, n=13 HCs; LES, n=19 HCs, n=10 calyces) of the utricular epithelium of mice between postnatal day (P) 7 and P200 (n = 45 mice). Hair bundles were displaced by a stiff probe with step and sinusoidal waveforms. We compared mechanotransduction (MET) currents and receptor potentials (V_RP_) in type I and II HCs from homozygous knockout (*Kcna10*^−/−^) animals and their wildtype (*Kcna10*^+/+^) or heterozygote (*Kcna10*^+/−^) littermates. *Kcna10*^+/+^ and *Kcna10*^+/−^ data were pooled (“control”) for properties that did not differ significantly between them. We next recorded postsynaptic potentials from calyces while displacing type I HC bundles. Finally, we compared the vestibulomotor performance of *Kcna10*^−/−^ and control mice on several tasks: VOR, open field arena, balance beam, swimming, and rotarod.

### K_V_1.8 channels reduce receptor potential gain and latency

**Figure 1.**
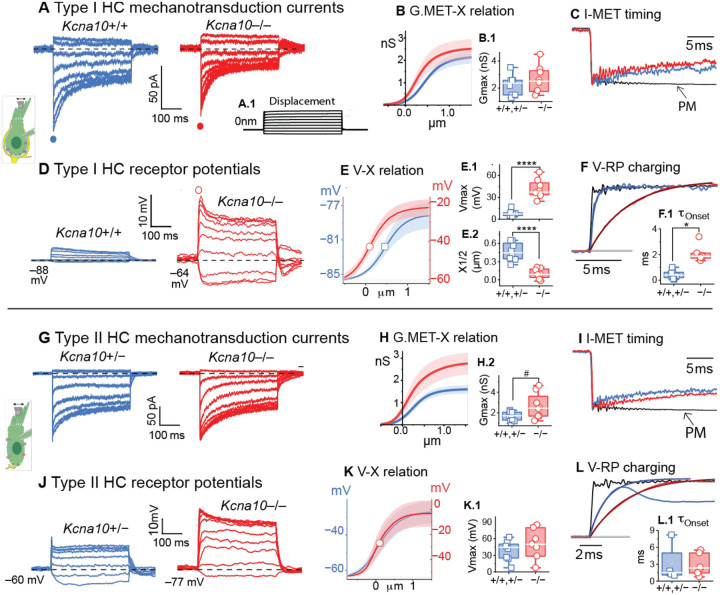
*Kcna10*^−/−^ hair cells have larger but slower receptor potentials. (**A**) Type I HCs with step-evoked MET currents across genotypes. ***A.1***, displacement stimulus (−0.3 to 1.2 μm) applies to entire figure. Exemplar MET currents from *Kcna10*^+/+^ at −87 mV, LES P17; *Kcna10*^−/−^ at −84 mV, MES P45. (**B**) Average of Eq.1 and 2 fits to peak I-X relation from *Kcna10*^+/+,+/−^ (n=8) and *Kcna10*^−/−^ (n=8) type I HCs. Shaded SEM. (**C**) Normalized current responses overlaid with +0.5 μm displacement showing onset time course <1 ms and adaptation. Probe motion (*PM*) measured from offline photodiode recording (see Methods). (**D**) *Kcna10*^−/−^ type I HCs produced larger but slower receptor potentials (V_RP_). Exemplar V_RP_ from same cells as B. (**E**) Average of Eq.1 fits to peak V_RP_(X) from *Kcna10*^+/+,+/−^ (n=8) and *Kcna10*^−/−^ (n=7); *open symbols*, X_1/2_; *arrow*, left-shifted X_1/2_. ***E.1***, V_Max_, t-test p=6E-6, g 3.6. ***E.1***, X_1/2_, ANOVA p=7E-5, g 3. (**F**) Voltage responses overlaid with +0.25 μm displacements and exponential fit (Eq. 3). ***F.1***,τ, KW p=0.02, g 1.6. (**G**) Type II HCs’ step-evoked I-MET obtained at −84 mV from *Kcna10*^+/−^, P20 LES; *Kcna10*^*−/−*^, P6 LES. (**H**) Average of Eq. 2 fits to peak G-X relation from *Kcna10*^+/+,+/−^ (n=8) and *Kcna10*^−/−^ (n=8). ***H.2***, G_Max_, ANOVA p=0.053, g 1.2, power 0.6. (**I**) Normalized current responses overlaid with +0.5 μm displacement showing onset time course <1 ms and adaptation. (**J**) Exemplar V_RP_ in type II HCs. (**K**) Peak V_RP_(X) averaged across cells. (**L**) Voltage responses to +0.25 μm displacements fit to Eq. 3. ***L.1***, *τ*, KW p=0.7, power 0.1. Full statistics in Table 1. Symbols: # p<0.1, *p<0.05, **** p<01E-5.

Transduction was stimulated by pushing a calibrated stiff probe against the back of a hair bundle in its preferred direction. When step displacements open mechanotransduction (MET) channels at the tips of the hair bundles, inward MET currents (I_MET_) flow and subsequently adapt, or desensitize, by up to 70% (Fig. 1A). MET currents were similar in control and *Kcna10*^−/−^ type I and II HCs (Fig. 1). MET currents were converted to conductance (G) to control for differences in holding potential across cells, and G_MET_(X) relations had an activation midpoint (X_1/2_) ~300 nm (Table 1, Eq. 1, Fig. 1B, H). MET currents activated in synchrony with the probe step and currents declined (adapted) with kinetics described by a double exponential (Fig. 1C). Genotype differences were not detected in maximal G_MET_, adaptation kinetics, X_1/2_, displacement sensitivity (slope, S), or resting open probability (P_O_).

Absence of K_V_1.8 strongly altered the magnitude and time course of receptor potentials. Resting membrane potential (V_rest_) was more hyperpolarized in control than *Kcna10*^−/−^ type I HCs (Fig. 1D) because g_K,L_ drives V_rest_ closer to E_K_. Control type I HCs produced small, fast receptor potentials that closely resembled the displacement step, because open g_K,L_ channels minimize input resistance at baseline (*V* = *IR*, Fig. 1D), reducing receptor potential gain and so minimizing distortions from voltage-dependent changes in current during the step stimulus. In contrast, *Kcna10*^−/−^ type I HCs produced receptor potentials that were initially quite large due to high input resistance and then relaxed as delayed rectifier K channels opened in response to the large depolarization (Fig. 1D).

Receptor potential-displacement curves (Fig. 1E) show differences in gain, V_Rest_, and X_1/2_ between control and *Kcna10*^−/−^ type I HCs. X_1/2_ was left-shifted in *Kcna10*^−/−^ type I HCs where larger displacement-evoked depolarizations resulted in the activation of delayed rectifier K_V_ channels, which acts to limit further depolarization. In contrast, g_K,L_, by attenuating the voltage changes, tuned the linear region of the sigmoidal V-X curve to larger (~0.5 μm) displacements, without significantly affect slope factor (dX, Table 1). These X_1/2_ data show how g_K,L_ not only reduces response time and increases the linearity of the receptor potential, but also shifts the operating range to larger stimuli. In Figure 1F, the role of g_K,L_ to reduce response latency is evident in the rapid V_RP_ charging time (membrane time constant, *τ*_*m*_) in control type I HCs, in which V_RP_ occurred as fast as the stimulus, while high *τ*_*m*_ slowed V_RP_ in *Kcna10*^−/−^ type I HCs.

The properties of type II HC MET currents (Fig 1G–I) were generally similar to those of type I HCs, at least in the extrastriolar cells studied here. On average, G_MET_ was higher in *Kcna10*^−/−^ type II than control type II HCs, bordering on significance (p=0.053) but with a large effect size (g=1.15). Given that *Kcna10*^−/−^ and control type II HC datasets did not differ by age, sex, and zone (Table 1), it’s possible *Kcna10*^−/−^ type II HCs exhibited a compensatory change, such as an increase in number of MET complexes inserted.

Control type II HCs produced receptor potentials with an initial sharp positive-going peak that relaxed as g_A_’s rapid activation and a decaying driving force repolarized the membrane towards equilibrium (Fig. 1J)^32^. *Kcna10*^−/−^ type II HCs produced receptor potentials that were larger because of higher input resistance and relaxed more slowly (Fig. 1J–K). In Figure 1L, control V_RP_ had shorter rise times because of lower input resistance and repolarized more quickly because g_A_ has faster activation kinetics than the residual K_V_7 delayed rectifier channels in *Kcna10*^−/−^ type II HCs^23^.

### *Kcna10*^−/−^ HC receptor potentials had lower lowpass corner frequencies

To see how the large differences in step-evoked responses affected frequency tuning, we displaced bundles with sinusoids incremented from 2 Hz to 100 Hz, spanning the upper range of natural head motion frequencies in mice (up to ~30 Hz)^33^. MET currents was similar across HC type and genotype (Suppl. Fig. 2); peak-to-peak gain increased with frequency, and, from 2–100 Hz, phase dropped from a lead of 20° to 0° (in phase).

**Figure 2.**
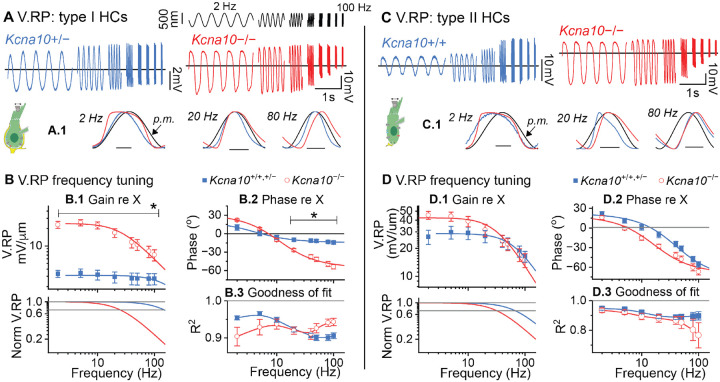
*Kcna10*^−/−^ hair cell receptor potentials have higher gains and lower lowpass corner frequencies. *Top panel*, displacement: bursts of sinusoid cycles from 2–100 Hz. (**A**) Representative type I HC responses (average of 6–13 presentations) to bundle displacement. *Left*, *Kcna10*^+/−^ P20 LES; reference line at −84.5 mV. *Right*, *Kcna10*^−/−^ P45 MES; reference line at −52 mV. (**A.1**) Normalized V_RP_ sinusoidal cycle overlaid with the probe motion (black). *Filled arrowhead*, probe motion (p.m.) measured from offline tracking with a photodiode (see Methods). *Scale bars, left to right:* 100 ms, 15 ms, 3 ms. (**B**) Type I HC V_RP_ referenced to X as functions of frequency; mean ± SEM values. (**B.1**) Peak-to-peak gain fit with Eq. 4; *bottom panel*, normalized to maximal AC gain parameter from fit. (**B.2**) Phase fit with Eq. 5. *, p<0.05 from posthoc pairwise comparisons in mixed ANOVA (Table 3). (**B.3**) Goodness-of-fit between responses and sinusoids is a measure of the linearity (fidelity) of representation of stimulus waveform. *Line*, spline. (**C**) Representative type II HC responses to bundle displacement. *Left*, *Kcna10*^+/+^ P22 MES; reference line at −57 mV. *Right*, *Kcna10*^−/−^ P49 MES; reference line at −52 mV. (**C.1**) Normalized V_RP_ sinusoidal cycle overlaid with the probe motion (black). *Scale bars, left to right:* 100 ms, 15 ms, 3 ms. (**D**) Type II HC V_RP_ referenced to X as functions of frequency; mean ± SEM. (**D.1**) Peak-to-peak gain fit with Eq. 4; *bottom panel*, normalized to maximal gain. (**D.2**) phase fit with Eq. 5. (**B.3**) Goodness-of-fit between responses and sinusoids; *line*, spline. Fit parameters in Table 3.

V_RP_ frequency tuning depended strongly on HC type and genotype. V_RP_ magnitude was approximately constant across frequency in control type I HCs but dropped over the same range in *Kcna10*^−/−^ HCs (Fig. 2A–B) and type II HCs (Fig. 2C–D). In Figure 2A.1, a close examination of each response cycle reveals that the *Kcna10*^−/−^ V_RP_ was distorted (square-like, not sine-like) at 2 Hz and lagged the stimulus >20 Hz. To further analyze the frequency dependence and faithfulness to the stimulus, we fit each burst of 5 cycles to a sine function; as expected, the goodness of fit was frequency-dependent (Fig. 1B.3, D.3).

We estimated *f*_LP_ by fitting V_RP_/X-Frequency curves with a lowpass filter transfer function (Eq. 4). *f*_LP_ was >100 Hz in control type I HCs and 14 Hz in *Kcna10*^−/−^ type I HCs (Fig. 2B.1). We also fit Phase-Frequency curves with a transfer function (Eq. 5); phase lag was much larger in *Kcna10*^−/−^ type I HCs above 10 Hz (Fig. 2B.2).

In control type II HCs, *f*_LP_ was 70 Hz, which dropped to ~20 Hz in *Kcna10*^−/−^ type II HCs (Fig. 2D.1). In Figure 2C.1, a close examination of each response cycle reveals that, at 20 Hz, only the control V_RP_ had a skewed response with a prominent peak that then relaxed. This skew was observed earlier in step-evoked responses (Fig 1L) and other studies^34^; it occurs because fast-activating g_A_ channels open and rapidly repolarize the membrane *after* V_RP_ has charged. *Kcna10*^−/−^ type I and II HCs had V_RP_ with similar lowpass tuning, reflecting their similar K_V_7 delayed rectifiers^23^.

### Synaptic transmission at *Kcna10*^−/−^ calyces

Based on the K^+^ current-driven nature of nonquantal transmission from HCs to calyces, we asked whether nonquantal postsynaptic responses were slower, smaller, or absent in calyces of *Kcna10*^−/−^ mice. We first characterized the intrinsic properties of calyces and did not detect differences between control and *Kcna10*^−/−^ calyces in their passive properties (R_in_, V_m_, C_m_), firing patterns elicited by current injection, or voltage-gated currents (I_KLV_, I_H_) (Suppl. Fig. 4, Suppl. Table 1), indicating similar baseline conditions and an absence of noticeable compensation.

**Figure 3.**
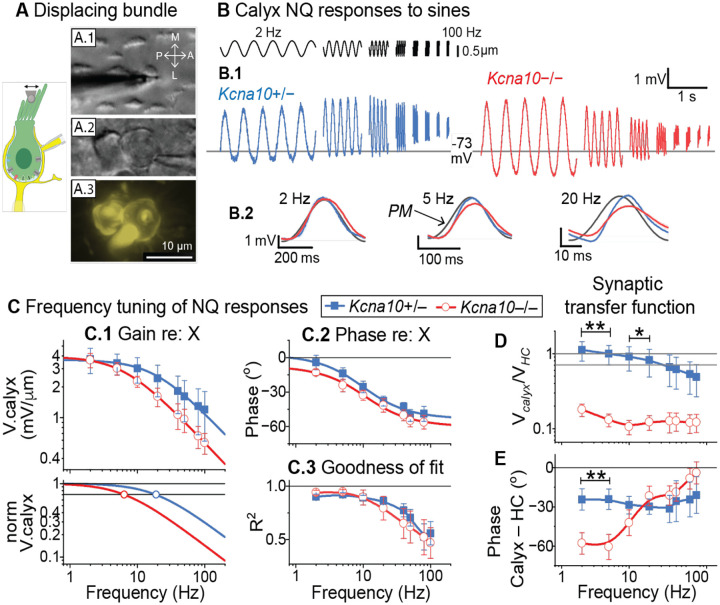
Non-quantal transmission from the type I HC to its calyx terminal differs between control and *Kcna10*^−/−^ utricles. (**A**) Afferent arbor bearing two calyces in the lateral extrastriola; stiff probe on the bundle of the type I HC (***A.1***, DIC) enclosed in the patched calyx (***A.2***); maximum intensity projection of the dye fill (***A.3***). (**B**) Sinusoidal type I HC bundle deflections (*top, black trace*) drove voltage responses (averages of 4–16 presentations) in extrastriolar calyces. (**B.2**) Population averages of sinusoidal responses at selected frequencies ± shaded SEM. PM, probe motion (*black*) from offline tracking with photodiode (see Methods). **(C**) Calyceal responses referenced to X as a function of frequency. (**C.1**) Peak-to-peak gain; population averages fit with Eq 4: *control*, magnitude 3.63 mV/μm, *f*_LP_ 18 Hz, n 1; *knockout*, magnitude 3.94 mV/μm, *f*_LP_ 6 Hz, n 1. *Bottom panel*: fit curves normalized to magnitude; open circles indicate when each curve crosses the −3dB line. (**C.2**) Phase; population average fit with Eq 5: *control*, steepness 36°, *f*_LP_ 9.9 Hz, baseline 3°; *knockout*, steepness 34°, *f*_LP_ 11 Hz, baseline −7°. (**C.3**) Goodness-of-fit between responses and sinusoids; *line*, spline. Mean ± SEM; control n=5, P52–105, median P99; null n=5, P15–220, median P37. (**D-E**) Isolation of the nonquantal synaptic transfer function by referencing calyx population average to type I HC population average. (**D**) Synaptic gain, V_Calyx_/V_HC_, ± SEM (Eq. 6a). (**E**) Synaptic phase (ϕ) lag, ϕ_Calyx_ – ϕ_HC_, ± SEM (Eq. 6b). *Line*, spline. *Asterisks*: *, p<0.05; **, p<0.01. Exact p-values in Table 3.

We measured nonquantal transmission by displacing the bundle of a type I HC while recording from its calyx in whole-cell patch clamp (Fig. 3A). Sinusoidal bundle stimulation evoked bidirectional voltage fluctuations in the calyx that closely resembled V_RP_ (Fig. 3B). Quantal waveforms were not observed, consistent with others’ observations of a reduction in type I HCs’ attached ribbons with development^35^.

As with HC responses, we analyzed frequency dependence and faithfulness to the stimulus by fitting each burst of 5 cycles to a sine function. The low-frequency (2Hz) AC gain was similar for control and *Kcna10*^−/−^ calyces (~4 mV/μm, Fig. 3C.1), but increasingly diverged at higher frequencies. We estimated *f*_LP_ for population averages by fitting Eq. 4. *f*_LP_ was higher in control (18 Hz, n=5) than *Kcna10*^−/−^ calyces (6 Hz, n=5, Fig. 3C.1).

We next asked whether the knockout’s effect on *f*_LP_ could be attributed solely to type I receptor potentials or also to differences in K^+^-mediated synaptic transmission. We isolated the synaptic transfer function by referencing the population average of calyx responses (n = 5, LES; controls, P58–105; *Kcna10*^−/−^, P15–220) to the population average of V_RP_ for type I HCs (control: n = 8 (6:2 LES:MES), P7–28; *Kcna10*^−/−^: n = 7 (1:6 LES:MES, P15–60; different experiments). Synaptic gain was near unity in controls, and much lower in *Kcna10*^−/−^ animals (Fig. 3D.1), where V_RP_ is large. Phase lag was lower below 10 Hz (Fig. 3D.2) and less frequency dependent; *Kcna10*^−/−^ phase lag decreased with frequency. While not statistically significant, these phase and gain differences suggest an explanation for the consequences for vestibular-driven reflexes and behaviors.

### Vestibulo-ocular reflexes in *Kcna10*^−/−^ mice have reduced gain and more variable phase

**Figure 4.**
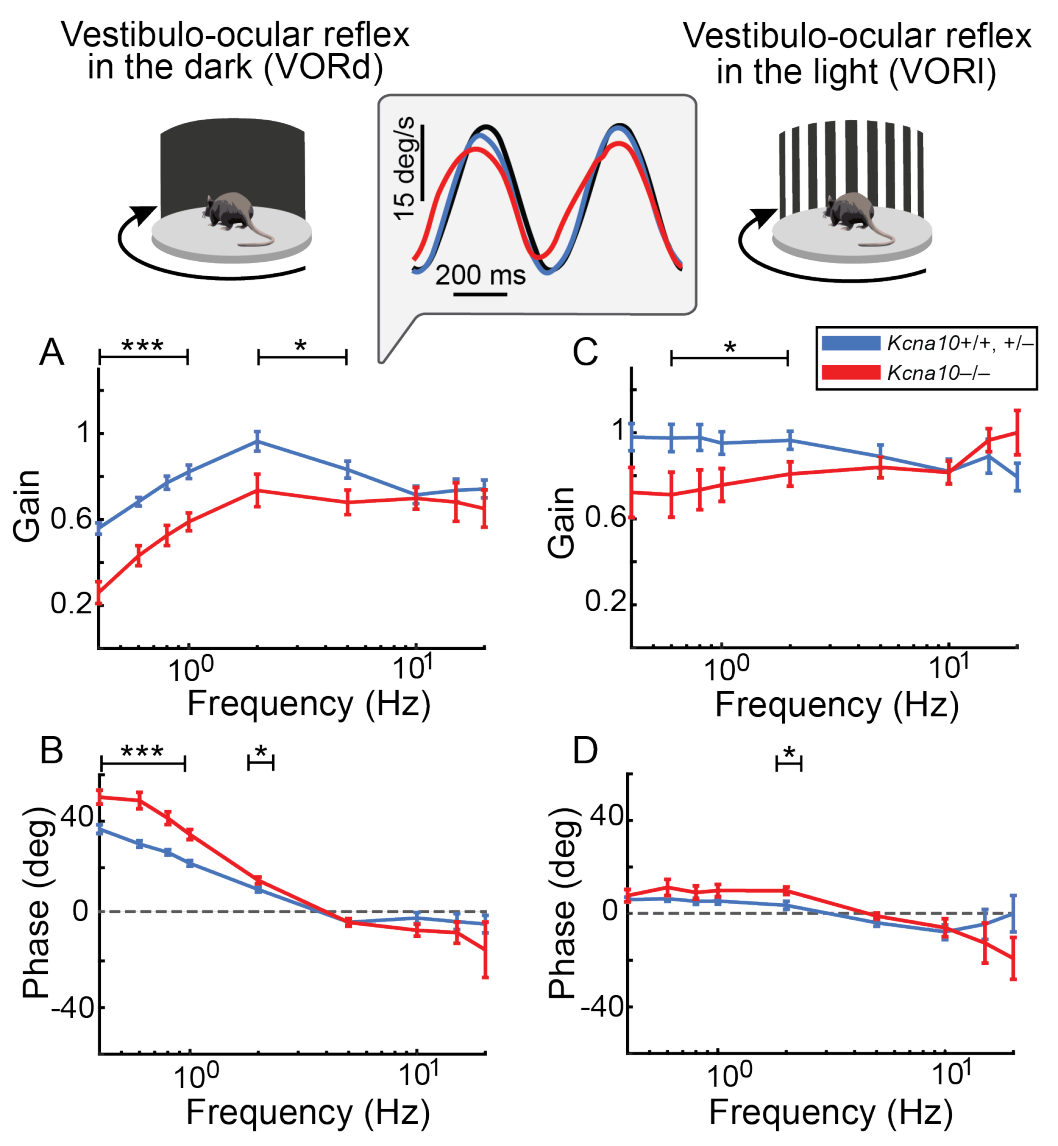
Vestibulo-ocular reflexes in *Kcna10*^−/−^ mice exhibit reduced gain and more frequency-dependent phase. **(A, B)** Vestibulo-ocular reflex in the dark (VORd) data for *Kcna10*^+/+,+/−^ mice (blue, N = 14) are compared with *Kcna10*^−/−^ mice (red, N = 9). VORd gains at 0.4, 0.6, 0.8, 1, 2, and 5 Hz were significantly reduced in *Kcna10*^−/−^ mice compared to *Kcna10*^+/+,+/−^ mice. VORd phases at 0.4, 0.6, 0.8, 1, and 2 Hz deviated significantly further from the ideal 0° phase in *Kcna10*^−/−^ mice. (**C, D)** Vestibulo-ocular reflex in the light (VORl) data for *Kcna10*^+/+,+/−^ mice (blue, N = 12) are compared with *Kcna10*^−/−^ mice (red, N = 9). VORl gains at 0.6, 0.8, 1, and 2 Hz were significantly reduced in *Kcna10*^−/−^ mice and deviated further from 0° phase at 2Hz in *Kcna10*^−/−^mice. **Inset:** Example traces of head velocity (black) and eye velocity for *Kcna10*^+/+,+/−^ mice (blue) and *Kcna10*^−/−^ mice (red) at 1 Hz in the dark. Eye velocities were inverted for comparison with head velocities. Mean ± SEM. *Asterisks*: *p < 0.05, ***p < 0.001. Statistics in Suppl. Table 2–3.

HC-afferent synapses are the first site of the 4–5 synapse VOR relay, which loops through the vestibular nucleus and commands ocular muscles to rotate *equal and opposite* to a detected head motion. The VOR is remarkably fast—occurring in just 5–6 ms^30^. To see if and how changes in inner ear signals in *Kcna10*^−/−^ animals affect VOR, we measured horizontal angular VOR responses in *Kcna10*^−/−^ mice and their control counterparts by rotating the mouse horizontally while a platform-mounted camera tracked pupil movement. VORs were measured in the light (VORl), where vision contributes, and the dark (VORd). Up to 5 Hz, VORd gain was closer to 1 in controls (ideal gain = 1, i.e. eye movements are equal in magnitude to the imposed head motion, Fig. 4A) than *Kcna10*^−/−^ mice. Lower gain in *Kcna10*^−/−^ mice indicates that they under-compensated for the imposed head motion. Below 5 Hz, VORd phase was closer to 0° in control than in *Kcna10*^−/−^ mice (ideal phase = 0°, i.e. eye movements match the timing of the imposed head motion, Fig. 4B). The greater phase lead at low frequencies in *Kcna10*^−/−^ mice means that eye rotations occurred too quickly. The greater frequency dependence of VOR phase in *Kcna10*^−/−^ mice resembles the knockout effect on phase of the type I HC V_RP_ (Fig. 2B.2).

We also observed that *Kcna10*^−/−^ mice had lower VORl gain (Fig. 4C), which has been noted in another model of vestibular dysfunction^36^. Moreover, the advanced age of the VORl cohort (median: 18 months) may have increased reliance on vestibular input in the light as retinal function declines in C57B6 mice started around 12–18 months of age^37,38^.

### *Kcna10*^−/−^ mice have specific deficits on challenging balance tasks

We next assessed whether a loss of K_V_1.8 could be directly linked to differences in performance during high-demand vestibular-associated tasks in mice aged 2–12 months (Suppl. Table 2–7). *Kcna10*^+/+^ and *Kcna10*^+/−^ littermates were pooled as controls when there was no detectable difference between them on a given task. *Kcna10*^−/−^ mice were healthy, developed normally, and did not display overt signs of vestibular dysfunction such as circling and head tilt (Suppl. Fig. 4A–B)^24^. K_V_1.8 is not expressed in neural tissue, but some reports in skeletal muscle and kidney^24^ led us to first characterize behavior on general motor and motor learning tasks. Both *Kcna10*^−/−^ and control mice successfully learned a rotarod task in the light (Suppl. Fig. 4C, Suppl. Table 2), and had no detectable differences in grip strength, gait, and most behaviors in an open arena (Suppl. Fig. 4D, Suppl. Table 4–5), except for rearing.

**Figure 5.**
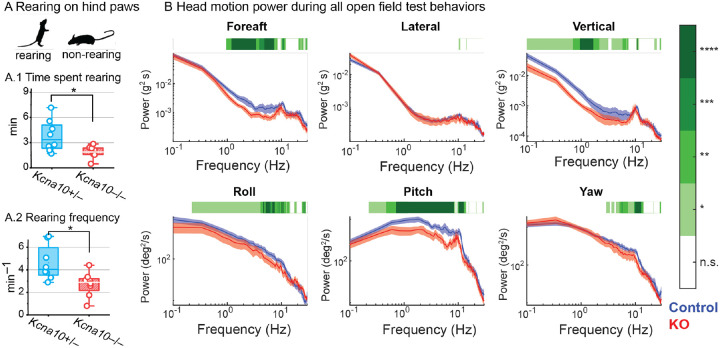
*Kcna10*^−/−^ mice are less mobile in an open field. (**A**) Time spent rearing (***A.1***) and rearing frequency (***A.2***) during a 1-hr exploration of an arena. The mouse’s position in the vertical and horizontal axes was detected by infrared beam breakage. No differences were detected in overall activity and centrophobism between *Kcna10*^+/−^ (n=8) and *Kcna10*^−/−^ (n=8) mice (**Table 4**). (**B**) Head motion power spectra in the translational (top panel) and rotational (bottom panel) during a 15-minute exploration in *Kcna10*^+/+,+/−^ (n=11) and *Kcna10*^−/−^ (n=6) mice. Asterisks: * p<0.05, ** p<0.01, *** p<0.001, **** p<0.0001. Mean ± shaded SEM.

In an open arena, *Kcna10*^−/−^ mice were less likely to rear up on their hindlegs, a normal exploratory bipedal behavior (Fig. 5A). The reduction in rearing seemed unrelated to anxiety or fear as *Kcna10*^−/−^ mice had normal centrophobism and overall activity (Suppl. Table 5). We quantified finer details of head control by tracking head motion with head-mounted inertial measurement units (IMU) and comparing the power spectra between control and *Kcna10*^−/−^ animals. *Kcna10*^−/−^ mice had lower head motion power in fore-aft (utricle), lateral (utricle and saccule), and vertical (saccule) translations, as well as roll and pitch (semi-circular canals, Fig. 5B). Lower pitch power was likely linked to reduced rearing, and avoidance of unstable bipedal positions.

**Figure 6.**
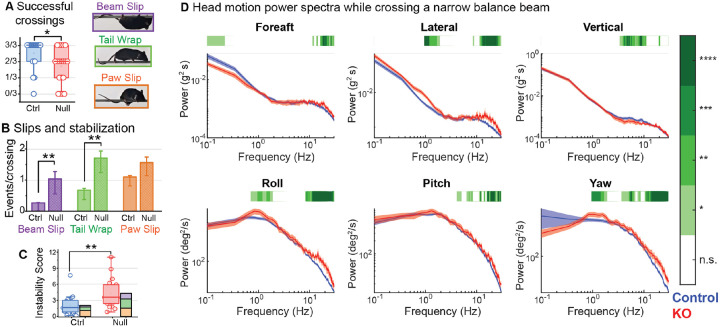
*Kcna10*^−/−^ mice have difficulty crossing a narrow balance beam. (**A**) Fraction of trials on which mice crossed without requiring rescue (*Kcna10*^+/+,+/−^, n=24; *Kcna10*^−/−^, n=19). (**B**) Per trial occurrences of beam slips, tail wraps, and paw slips per mouse were summed as an Instability Score (***C***) to account for individual variability. Statistics in Table 3. (**D**) Head motion power spectra in translational (top row) and rotational (bottom row) dimensions in *Kcna10*^+/+,+/−^ (n=11) and *Kcna10*^−/−^ (n=7) mice. Mean ± shaded SEM. *Green bars at the top* show data ranges that differ statistically; see *color map on right*. *Asterisks*: * p<0.05, ** p<0.01, *** p<0.001, ****p<0.0001.

*Kcna10*^−/−^ mice were less stable while crossing a narrow balance beam. Most control mice (83%) but only half of *Kcna10*^−/−^ mice (56%) mice crossed successfully without requiring rescue after slipping off the beam (p<0.01, Fig. 6A). *Kcna10*^−/−^ mice were more likely to slip off the narrow balance beam and compensated by more frequently wrapping their tails around the beam for additional stability (Fig. 6B). On the beam, *Kcna10*^−/−^ mice had more head motion power >15 Hz in all dimensions except vertical translation (Fig. 6D, top right).

**Figure 7.**
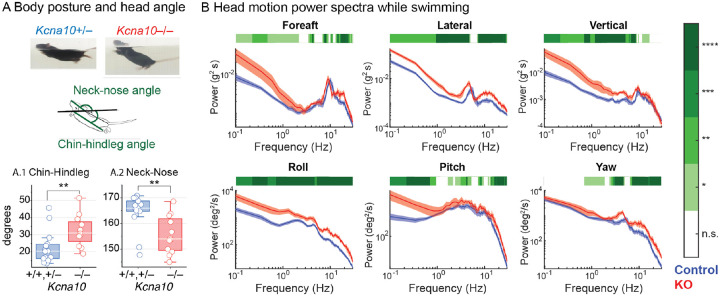
*Kcna10*^−/−^ mice swim at a higher angle relative to the water surface and with extra head motion. (**A**) Body posture and head tilt during a 30-second swim in *Kcna10*^+/+,+/−^ (n=15) and *Kcna10*^−/−^ (n=9) mice. (**A.1**) Body posture measured as the angle between the chin-hindleg line and the waterline. (**A.2**) Head pitch measured as the angle between the neck-nose line and the waterline. Statistics in Supplemental Table 7. (**B**) Head motion power spectra in translational (top row) and rotational (bottom row) dimensions in *Kcna10*^+/+,+/−^ (n=11) and *Kcna10*^−/−^ (n=6) mice. Mean ± shaded SEM. *Green bars at the top* show data ranges that differ statistically; see *color map on right*. *Asterisks*: * p<0.05, ** p<0.01, *** p<0.001, **** p<0.0001.

In the water, *Kcna10*^−/−^ mice stayed afloat but swam less effectively, with a more vertically tilted body posture and head angle (Fig. 7A). While swimming, *Kcna10*^−/−^ mice had more head motion power (more thrashing) in all dimensions and over a wide range of frequencies (Fig. 7B). This lack of coordination was especially prominent in the water, where normal proprioceptive cues are not present to compensate for vestibular deficits^39^.

Overall, these results suggest that K_V_1.8’s effects on receptor potentials and downstream synapses and circuitry support effective gaze stabilization, stability in difficult terrestrial balance maneuvers, and postural control in the water.

## Discussion

K_V_1.8 expression is necessary for the major K_V_ conductances of both type I and type II HC membranes^23^. Here we show that in the extrastriolar zone of the mouse utricle, K_V_1.8 reduces response latency and extends the frequency range to higher frequencies for receptor potentials of both hair cell types and for signals transmitted across the unusual type I HC-calyx synapse. Further, knocking out K_V_1.8 increased distortion (nonlinearity) of the representation of displacement stimulus at low frequencies, and, in type I HCs, shifted the operating range to smaller displacements. Knocking out K_V_1.8 impaired or reduced multiple vestibulomotor behaviors: VOR, voluntary engagement in unstable exploratory behaviors, balancing on a narrow beam, and posture in the water. The absence of observable effects on calyx intrinsic properties or non-balance motor tasks leads us to conclude that K_V_1.8 contributes to vestibular function at the level of the hair cell by increasing its frequency range, prioritizing linear detection of large stimuli, and supporting non-quantal transmission.

### K_V_1.8 and signal processing (receptor potentials and synaptic transmission)

Given the low input resistance imparted by g_K,L_ and its hyperpolarized activation range, control type I HCs have low gains and fast membrane charging times. Low gain attenuates the extent to which bundle displacements activate voltage-dependent channels, which may distort or compress the waveform, and as a result increases the linearity of the receptor potential. These findings build on previous current injection experiments by demonstrating the same principles in receptor potentials, which additionally process the nonlinearities of MET currents as the HC would during a head motion. Further, our receptor potential recordings revealed that g_K,L_ (K_V_1.8) shifts the operating range to larger stimuli (by ~0.5 μm).

In the frequency domain, fast membrane charging times give type I HC receptor potentials a flat, frequency-independent gain up to 100 Hz, with phase close to 0° throughout. The ability to respond linearly and consistently to large head motions over a broad frequency range are major effects of g_K,L_ on the type I receptor potential, which shapes downstream signals in the afferent. By participating in NQ transmission, which is faster and more linear than quantal transmission, g_K,L_ further contributes to the bandwidth and linearity of the afferent response (see below).

In type II HCs, by contributing to K^+^ conductances that open after depolarization positive to rest, K_V_1.8 enacted more left-shifted V-X and V-Frequency relations than in type I HCs. Because R_in_ was initially large and then shrank with time and displacement magnitude, receptor potentials had a forward skew and left-shifted X_half_. Although the effect of knocking out K_V_1.8 on *τ*_Onset_ of receptor potentials did not rise to the level of significance, our previous experiments with injected current were more powered—owing to lower biological variability and higher throughput—and clarify that knockout of K_V_1.8 does increase *τ*_RC_ in type II HCs^23^. In the frequency domain, the ‘forward skew’ of the receptor potential in the knockout hair cells presented as high phase lead at low frequencies. Phase and gain dropped precipitously at higher frequencies as the MET current outsped K_V_ channels and *τ*_RC_.

We show, for the first time, that g_K,L_-null HCs can drive nonquantal transmission at the type I-calyx synapse. Nonquantal signals in control and *Kcna10*^−/−^ calyces had similar gains relative to probe motion at 2 Hz, despite the large difference in receptor potential magnitude — illustrating that in the control situation, K_V_1.8 effects on NQT compensate for the small type I HC receptor potential, meaning that the control synapse is more efficient even at low frequencies. At higher frequencies, the advantages conferred by K_V_1.8 become more obvious: *Kcna10*^−/−^ calyces had lower *f*_LP_ and more phase lag (slower responses), consistent with their long charging times in response to step displacements. *Kcna10*^−/−^ calyceal responses were slower both because of the high starting input resistance and because the residual, K_V_7-mediated delayed rectifier channels require time to open during receptor potential^23^, whereas g_K,L_ has a high open probability at resting potential, as shown for these particular cells^8^.

In both control and *Kcna10*^−/−^ calyces, we observed substantial lowpass filtering in the synaptic transfer function; that is, regardless of genotype, *f*_LP_ was lower in calyces (*control*, ~18 Hz; *null*, ~6 Hz) than in type I HCs (*control*, >100 Hz; *null*, ~20 Hz). We attribute some of this lowpass filtering to the calyx’s own membrane time constant, an estimated 1.2 ms (Suppl. Table 1), which corresponds to a theoretical *f*_LP_ of 110 Hz. The low-voltage activated K channels on the calyx side of the synapse have less negative activation ranges than g_K,L_, and thus greater initial input resistance, and the calyx membrane is in series with a long neurite, reducing their electrical input further.

Previously, we have reported non-quantal transmission with higher frequency tuning^5^. In that study, Songer recorded subthreshold nonquantal responses with *f*_LP_ = ~70 Hz, much higher than the 18 Hz value in this study (our Fig. 3C.2). Multiple meaningful differences between Songer’s and our experiments could account for the discrepancy: organ (*Songer*, saccule; *here*, utricle), epithelial zone (striola *vs*. lateral extrastriola), calyx type (higher proportion of double calyces in saccule), displacement intensity (0.3–1 μm; 1 μm), species (rat; mouse), and age (P4–P9; P12–202). Organ and zone are likely the most potent differences. The striola of mammalian otolith organs is specialized for high frequency stimuli: both saccular and utricular striolas respond to bone-conducted vibrations^40^, and saccular striolar afferents respond to sounds^41^ and drive the VsEP^42–44^. The VsEP is extremely rapid (latency ~80 μs^45^) and represents the synchronized activity of striolar saccule afferents, yet is absent in *Kcna10*^−/−^ mice^24^. Our physiology from the extrastriolar utricle suggests that the missing VsEP reflects the inability to keep up with hair bundle deflections that we have presented here.

In this context, it may be worth noting that *Kcna10*^−/−^ mice share some deficits with *Cyp26b1*^−/−^ mice, which lack the striola and instead have normal extrastriolar HCs^44^ throughout the epithelium. Like *Kcna10*^−/−^ mice, *Cyp26b1*^−/−^ mice have absent VsEPs and abnormal head control. We will discuss this in the next section, where we interpret vestibulomotor behaviors through the lenses of single-cell physiology and downstream circuitry from different vestibular epithelia and epithelial zones.

### K_V_1.8 and vestibulomotor processing

While this work was also motivated by the mystery of the seemingly unique type I HC conductance (g_K,L_), we quickly learned that g_K,L_ is closely related, through the K_V_1.8 subunit, to g_A_ and part of g_DR_ in type II HCs. In the knockout, we are looking at the combined effects of K_V_1.8 across all vestibular HCs. The absence of general motor deficits in *Kcna10*^−/−^ mice suggests that vestibulomotor deficits occurred through the physiological effects of knocking out K_V_1.8 on HCs and HC-afferent transmission.

The horizontal angular VOR is driven by the lateral canal peripheral zone, which bears physiological similarities to the otolith extrastriolar zone but see^46^. In control mice, VORd gain was high (i.e. ~1) and phase was close to 0 whereas *Kcna10*^−/−^ mice produced suboptimal VORd with lower gain—despite higher V_RP_ gain and similar synaptic transmission gain—and phase that was more strongly frequency-dependent. Stronger frequency-dependence was also seen in the V_RP_ phase of *Kcna10*^−/−^ type I HCs. The knockout’s effect on low-frequency VORd may reflect the distorted (square-like) V_RP_ waveforms (Fig 2A.1, C.1, *2 Hz*).

On the balance beam, *Kcna10*^−/−^ mice had more head motion power >10 Hz, which, coupled with gaze fixation deficits, likely contributed to poor balance. *Kcna10*^−/−^ mice developed a compensatory balance strategy, tail wrapping, that has been observed in rod-crossing assays of deer mice^47^ but not previously reported in vestibular mouse mutants. The emergence of tail wrapping in *Kcna10*^−/−^ mice may reflect the moderate phenotype or constitutive nature of the knockout, which allowed mice 2+ months to learn compensatory strategies. Behavioral compensation was also observed in the open field arena, where *Kcna10*^−/−^ mice rarely assumed an unstable bipedal stance and moved their heads less, reminiscent of how some patients with vestibular inner ear pathologies tend to keep their heads still^48^.

The postural deficits of *Kcna10*^−/−^ mice in the water may also reflect K_V_1.8 knockout’s effect on type II HCs and thus quantal transmission. Type II inputs to afferents are more numerous in the extrastriola, and extrastriolar (regular) afferents are associated with signaling static head tilt re: gravity^49^. We observed seemingly normal righting reflexes in *Kcna10*^−/−^ pups (P5–P13), suggesting that gravity sensing is functional. Higher head motion power reflected thrashing movements in the swim patterns of *Kcna10*^−/−^ mice. Based on the slower speed of signal transmission in the *Kcna10*^−/−^ inner ear, this lack of coordination likely arose because vestibular feedback about self-motion was too slow or unreliable to drive effective motor coordination. This deficit was especially prominent in the water, where normal proprioceptive cues are not present to compensate for such vestibular deficits^39^. Since *Cyp26b1*^−/−^ mice, which lack a striola, have normal swim behavior^44^, we attribute the observed swim deficits to the impact of *Kcna10* deletion on the peripheral and extrastriolar zones.

### Significance

This work was motivated in part by the current lack of a broader understanding of NQT’s role in amniote vestibular function. The *Kcna10*^−/−^ mouse addresses this gap as one of the first genetic models that perturbs NQT at the type I HC-calyx synapse. This model, as well as others, may be leveraged to understand why NQT so robustly drives vestibular function, as evidenced by observations that pharmacological or genetic inhibition of glutamatergic transmission fails to ablate vestibular outputs^19,21^.

## Materials and Methods

### Preparation

All procedures for handling animals followed the NIH Guide for the Care and Use of Laboratory Animals and were approved by the Institutional Animal Care and Use Committees of the University of Chicago (Animal Care and Use Procedure #72360) and Johns Hopkins University (Animal protocol #MO23M106). All mice belonged to a transgenic line with a knockout allele of *Kcna10* (referred to here as *Kcna10*^−/−^). Our breeding colony was established with a generous gift of such animals from Sherry M. Jones and Thomas Friedman. These animals are described in their paper^24^. Briefly, the Texas A&M Institute for Genomic Medicine generated the line on a C57BL/6;129SvEv mixed background by replacing Exon 3 of the *Kcna10* gene with an IRES-bGeo/Purocassette. Mice in our colony were raised on a 12:12h light-dark cycle with access to food and water *ad libitum*.

Preparation, stimulation, and recording methods followed our previously described methods for the mouse utricle^50^. Mice (P6–259) were anesthetized through isoflurane inhalation. After decapitation, each hemisphere was bathed in ice-cold, oxygenated Liebowitz-15 (L15) media. The temporal bone was removed, the labyrinth was cut to isolate the utricle, and the nerve was cut close to the utricle. The utricle was treated with proteinase XXIV (100 mg/mL, ~10 mins, 22°C) to facilitate removal of the otoconia and attached gel layer and mounted beneath two glass rods affixed at one end to a coverslip.

### Electrophysiology

We used the HEKA Multiclamp EPC10 with Patchmaster acquisition software, filtered by the integrated HEKA filters (a 6-pole Bessel filter at 10 kHz and a 4-pole Bessel filter at 5 kHz), and sampled at 10–100 kHz. Recording electrodes were pulled (PC-100, Narishige) from soda lime glass (King’s Precision Glass R-6) and wrapped in paraffin to reduce pipette capacitance. Internal solution contained (in mM) 135 KCl, 0.5 MgCl_2_, 3 MgATP, 5 HEPES (2-[4-(2-hydroxyethyl)piperazin-1-yl]ethanesulfonic acid), 5 EGTA (ethylene glycol-bis(β-aminoethyl ether)-N,N,Nʹ,Nʹ-tetraacetic acid), 0.1 CaCl_2_, 0.1 Na-cAMP, 0.1 LiGTP, 5 Na_2_CreatinePO_4_ adjusted to pH 7.25 and ~280 mmol/kg by adding ~30 mM KOH. External solution was Liebowitz-15 media supplemented with 10 mM HEPES (pH 7.40, 310 ± 10 mmol/kg). Recordings were conducted at room temperature (22–25°C) to preserve the lifespan of valuable preps and to minimize the electrical noise and physical constraints of warming devices during challenging experiments. Pipette capacitance and membrane capacitance transients were subtracted online with Patchmaster software. Series resistance (8–12 MΩ) was measured after rupture and compensated 0–80% with the amplifier. Potentials are corrected for remaining (uncompensated) series resistance and liquid junction potential of ~+4 mV, calculated with LJPCalc software^51^.

Type I HCs with g_K,L_ were transiently hyperpolarized to −90 mV to deactivate g_K,L_ enough to increase *R*_in_ above 100 MΩ, as needed to estimate series resistance and cell capacitance. Control type I HCs’ average resting potential, V_rest_, was −89 mV ± 1 (8), unusually negative for a calculated E_K_ of −86.1 mV.

### Transduction

Hair bundles were deflected by a stiff probe (borosilicate, tip width <1 μm) attached to a piezoelectric planar bimorph. Driving voltage to the bimorph was lowpass filtered by an 8-pole Bessel filter (Active Tunable Filter Model 900, Frequency Devices, Ottawa, IL) at a corner frequency of 1000 Hz (resonant frequency = 2250 Hz). Probe time course was monitored offline by projecting the probe image onto the edge of a photodiode (PIN-6D). At the end of each experiment, steady-state probe tip distance per driving voltage was measured using a slow step stimulus, camera, and DIC illumination. In figures, all displacement traces are photodiode signals scaled to the known voltage sensitivity of the probe.

We evoked MET currents and V_RP_ by displacing the hair bundle along its sensitive axis with a stiff glass rod placed behind and approximately halfway up the hair bundle. Resting probe position was selected to be 10–20% of the operating range of the bundle, evoking 16 ± 2 % (n = 38) of maximum MET currents [percent P_o_ = 100*(I_X=0_ – I_min_)/(I_max_ – I_min_)]. We expect this imposed resting position to be slightly higher than natural resting position because we pushed the bundle slightly to ensure mechanical coupling with the probe. Most HCs were held at −84 mV to increase driving force on MET current and reduce noise from depolarization-activated channels. Some type I HCs were held negative to −84 mV to fully deactivate g_K,L_, which is so large as to degrade the voltage clamp. Some control type I HCs with g_K,L_ had MET currents with very slow rise times (Suppl. Fig. 1). We determined that the slow rise times were a consequence of low input resistance (open g_K,L_ channels) and poor voltage clamp (uncompensated series resistance). Slow rise times could be rectified by closing/blocking g_K,L_ channels or increasing series resistance compensation, which was replicated in simulations of a voltage-clamp circuit (Suppl. Table 8). Therefore, we marked very slow MET current rise times as a recording quality issue and excluded those type I HCs from analysis in the rest of this paper.

### Transduction Analysis

Data analysis was performed using OriginLab (Northampton, MA) and custom MATLAB scripts.

#### Fitting sensitivity and time course of transduction currents

*G-X curves*. Peak transduction currents were the greatest absolute difference between the baseline and step current. Current was converted to conductance (G) using a reversal potential of +2.6 mV^52^. Sigmoidal dependence of G-X curves was fit with a first-order (Eq. 1) or second-order Boltzmann equation (Eq. 2^53^).


(1) $$G(X)=G_{\text{min}}+\frac{G_{\text{max}}}{1+e^{\frac{X_{\text{half}}-X}{k}}}$$

(2) $$G(X)=G_{\text{min}}+\frac{G_{\text{max}}}{1+\left(\text{exp}\frac{X_{1}-X}{k_{1}}\right)*\left(1+\text{exp}\frac{X_{2}-X}{k_{2}}\right)}$$

X_1_ is the first order midpoint, k_1_ is the first order slope factor, inversely related to curve steepness at the midpoint, X_2_ is the second order midpoint, and k_2_ is the second order slope factor.

##### Adaptation time course.

Adaptation of transduction currents were fit with Eq. 2.


(3) $$I(t)=I_{SS}+A_{f}*\text{exp}\left(-\frac{t}{\tau_{f}}\right)+A_{s}*\text{exp}\left(-\frac{t}{\tau_{s}}\right)$$

*I*_*SS*_ is steady-state current, *A*_*f*_ and *A*_*s*_ are amplitudes of fast and slow adaptation, and *τ*_*f*_ and *τ*_*s*_ are time constants of fast and slow adaptation, respectively.

##### Lowpass filter.

We calculated the AC component of responses to sinusoidal bundle displacement by fitting each 5-cycle burst with sine waves and referencing the fit to bundle displacement, producing gain (pA/μm or mV/μm) and phase (degrees) relative to the stimulus. We fit gain with a simple lowpass filter (Eq. 4a, as in^54^) or a bandpass filter (Eq. 4b); the latter was applied to control type II hair cells where V_RP_ had a noticeable high pass component that we attribute to g_A_ inactivation. Fits to MET current gain were sometimes restricted to the 2–20 Hz range, where the effects of transducer adaptation dominate as a high-pass filter.

(4a) $$G(f)=\frac{G_{\text{max}}}{\sqrt{1+{\left(\frac{f}{f_{c}}\right)}^{2n}}}$$

(4b) $$G(f)=\frac{G_{\text{max}}}{\sqrt{\left(1+{\left(\frac{f}{f_{HP}}\right)}^{-1}\right)*\left(1+{\left(\frac{f}{f_{LP}}\right)}^{2}\right)}}$$

*f*_*c*_ is the corner frequency, and |*n*| is the order (pole) of the filter; *n* = −0.5 for MET currents and *n* = 1 for V_RP_ and postsynaptic potentials. |*n*| = 0.5 produced the best fit for MET currents, but it should be noted that theoretically poles should be integers. *f*_*HP*_ and *f*_*LP*_ are *f*_*c*_ for high-pass and low-pass transfer functions. Phase (*ϕ*) was fit with Eq. 5, which is conventional for a simple one-pole lowpass filter and also includes two additional terms (*a*, *b*) to improve the fit. *a* is the steepness of the phase-frequency relation and *b* is the baseline phase.


(5) $$\phi (f)=-a*\text{arctan}\left(\frac{f}{f_{LP}}\right)+b$$

##### Synaptic transfer function.

Synaptic gain was calculated as the ratio of the population averages of displacement-evoked potentials in calyces and type I HCs, or $\left({\bar{x}}_{\text{Calyx}},{\bar{x}}_{HC}\right)$. SEM $\left(\sigma_{\bar{x}}\right)$ of synaptic gain was estimated from the means $\left({\bar{x}}_{\text{Calyx}},{\bar{x}}_{HC}\right)$ and standard errors $\left({\bar{x}}_{\text{Calyx}},{\bar{x}}_{HC}\right)$ using the delta method (Eq. 6a). The covariance term was set to 0 because calyx and type I HC recordings were obtained from different preparations.


(6a) $$\sigma_{\bar{x}}={\left({{\bar{x}}_{HC}}^{-2}\left({\sigma_{\bar{x},\text{Calyx}}}^{2}+{\left({\bar{x}}_{\text{Calyx}}/{\bar{x}}_{HC}\right)}^{2}{\sigma_{\bar{x},HC}}^{2}\right)\right)}^{1/2}$$

Synaptic phase (*ϕ*) lag was calculated as the difference between the population averages of AC phase, or ${\bar{\phi }}_{\text{Calyx}}-{\bar{\phi }}_{HC}$. SEM $\left(\sigma_{\bar{x}}\right)$ of synaptic phase lag was the root sum of squares (Eq. 6b).


(6b) $$\sigma_{\bar{x}}={\left({\sigma_{\bar{x},HC}}^{2}+{\sigma_{\bar{x},\text{Calyx}}}^{2}\right)}^{1/2}$$

### Behavior

Animals were introduced to the behavior room the day before experiments.

#### Balance Beam:

Animals were allowed at least 5 minutes to acclimate to the testing room. Mice were trained for 3 trials on a wide beam (12 mm wide), given 10 minutes of rest, and then tested for 3 trials on a narrow beam (6 mm wide). To incentivize mice to cross the flat beam, a bright lamp was put at the start of the beam, and a dark goal box (5 in × 5 in × 5 in) filled with food and bedding from their home cage was placed at the end of the beam. Mice were given at least 20 seconds to rest in the end box between trials. The beam was cleaned with Clidox-S between mice. Videos of the trial were taken at 30 frames per second (fps) with a lateral view of the beam. For manual scoring analysis, two genotype-blind scorers marked the following behaviors: “beam slip”, hanging upside down on the beam and needing rescue to finish (rescue time was included in total time to cross); “tail wrap”, wrapping the tail around the beam instead of having it in the air; and “paw slip”, a single paw slipping off the beam without the mouse falling off the beam. The crossing time was recorded as the time between the nose crossing the starting line and the nose crossing the finish line (64 cm). Crossing time included non-ambulatory epochs, including while the mouse was being rescued and while the mouse stopped and sniffed the air.

#### Swim test:

A clear aquarium (38×30×25 cm) was filled with warm water (30°C) and placed in front of a white background. Animals were placed directly into the water. Videos (30 fps) of the animal’s swim were recorded from the lateral side of the container, with the height aligned to the waterline. After 30 seconds, animals were retrieved, dried with paper towels, and placed in a cardboard box under a heat lamp. Videos were trimmed to the first section where the animal was swimming straight on the side closest to the camera. In eight random frames, two genotype-blind scorers marked body parts and calculated body angles in FIJI^55^.

#### Open field:

Animals were placed in open field boxes (ENV-510, 27 × 27 × 20.3 cm) with dim lighting (16 lux) to avoid excessive stress. After 10 minutes of acclimation, mouse movement was recorded for 1 h via infrared beam interruption. Activity Monitor 7 software (Med Associates, Inc, Fairfax, VT) returned data on the location of the mouse through time, and calculated Distance Travelled, Number of Ambulatory Episodes, Average Speed, Centrophobism, Jump Events, Rearing Events, and Rearing Time in 1-min bin sizes.

#### Rotarod:

The Columbus Instruments Rotarod was used with large dowels (diameter = 7 cm) and was cleaned with Clidox-S between mice. We used a 2-day protocol. On Day 1, there were 6–8 training trials wherein mice were initiated at 5 rpm before the ramp accelerated from 5 rpm to 44 rpm at a rate of 2.4 rpm per 4 seconds, with 30 sec rest between trials. On Day 2, the same protocol was used, with 4 refresher trials, a 20-minute break, and then 3 test trials. This 2-day protocol, which is shorter than some other rotarod protocols, is based on other studies of vestibular function^56–58^.

#### DigiGait:

Mice were placed on a treadmill-like apparatus with a clear belt and an internal camera, which records the ventral view of a mouse as it runs on the belt. Mice were recorded in a slow (15 cm/s) and fast (25 cm/s) trial, and the belt was cleaned with Clidox-S between mice. For analysis, in DeepLabCut, bodyparts were labelled for 35–70 frames per mouse for both the slow and fast trials. These labelled frames were used to train a DeepLabCut model in a Google Colab environment, which then applied labels to all frames. We wrote custom Matlab functions to analyze gait parameters from these body part positions based on published equations^59^.

#### Instrumented Behavioral Assessments:

To quantify the angular rate and linear acceleration of the head during open field, swim, and balance beam assessments, a miniature inertial measurement unit (IMU) was affixed to the mouse’s head as previously described^60,61^. For the open field and swim assessments, mice performed the tasks as described above, now with an IMU affixed to the head, three times at a 1-minute duration with a 1-minute inter-trial-interval. For balance beam, mice performed the task as described above in its entirety, now with an IMU affixed to the head.

#### Vestibulo-ocular reflex:

To perform vestibulo-ocular reflex (VOR) testing, mice were head-restrained using a surgically implanted custom headpost, as previously described^62^. The mice were secured to a motion platform (BREVA Dynamique - SN 21135, SYMETRIE) surrounded by a visual environment consisting of black and white stripes with a visual angle width of 5°, as described^62^. Eye positional movement data was collected using a platform-mounted camera (Firefly S USB3, FLIR) positioned ~10mm perpendicular to the mouse’s eye, sampled at 200 Hz. Custom pupil tracking code was used to determine the horizontal and vertical components of 3D eye positional movement. The velocity of the platform (head velocity) was collected with a MEMs inertial measurement unit (IMU) (ICM-46288-P, InvenSense/TDK) mounted on the mouse’s headpost, sampled at 500 Hz.

To evoke a VOR response, the motion platform applied rotational sinewaves at 0.4, 0.6, 0.8, 1, 2, 5, 10, 15, and 20 Hz with a peak velocity of ± 15 deg/s, tested under light and dark conditions. Synchronization pulses from IMU samples and camera frames were collected using NI Instruments systems (NI PXI-6133) and Spike GLX software at a 20kHz sampling rate. These pulses were used to synchronize the eye and head movement data, which were then resampled at 1kHz for analysis in MATLAB 2022b (Mathworks).

To compute the gain and phase of the VOR responses, eye position signals were differentiated to obtain eye velocity data. Segments of the eye movement data containing saccades or quick phase movements were identified and excluded from the analysis. Eye and head signals were low-pass filtered with a fourth-order Butterworth filter with a bandpass set at twice the stimulation frequency plus one. A least-square optimization was performed to determine the gain and phase at each frequency (see^62^ for details). The estimated VOR gain and phase were then plotted as the mean for each group ± standard error of the mean (SEM).

### Statistics

We give means ± SEM for normally-distributed data, and otherwise, median and range. Data normality was assessed with the Shapiro-Wilk test for n<50 and the Kolmogorov-Smirnov test for n>50. To assess homogeneity of variance we used Levene’s test. With homogeneous variance, we used one-way ANOVA for genotype with the posthoc Tukey’s test. When variance was non-homogeneous, we used one-way Welch ANOVA with the posthoc Games-Howell test. For data that were not normally distributed, we used the non-parametric one-way Kruskal-Wallis ANOVA (KWA) with posthoc Dunn’s test. Effect size is Hedge’s g (g). For age dependence, we used partial correlation coefficients controlling for genotype and zone. Statistical groups may have different median ages, but all have overlapping age ranges.

For vestibulo-ocular reflex gain and phase values, comparisons were performed using two-way repeated measures ANOVA with post hoc Bonferroni’s corrections.

For power spectra analysis, we used an independent sample permutation test to test significant differences between the two groups.

## Supplementary Material

1

## Figures and Tables

**Table 1.** Step-evoked MET currents and adaptation in extrastriolar hair cells. Mean ± SEM (number of cells, if different from the set). *g*, Hedge’s g affect size; *pwr*, statistical power; *ST*, Student’s t-test; *A*, ANOVA (Tukey’s HSD); *W*, Welch ANOVA (Games-Howell test); *KW*, Kruskal-Wallis (Dunn’s test); *LM*, linear regression. Factors were not analyzed if there was *ID*, insufficient data; *UD*, unequal distribution.

					G(X) Boltzmann parameters				Adaptation at X1/2	
Type	*Kcna10*		n	Age (median)	Max G_MET_ (nS)	X_1/2_ (nm)	S (nm)	Po (%)	τ_F_ (ms)	τ_s_ (ms)
**I**	**+/+, +/−**		8	7–28 (18)	2.0 ± 0.3	340 ± 70	330 ± 70	17 ± 5	100 ±100	600 ± 300
	**−/−**		8	15–60 (45)	2.5 ± 0.3	230 ± 90	280 ± 60	20 ± 5	4.3 ± 2	400 ± 200
**WT (1) vs HET (7)**					ID	ID	ID	ID	ID	ID
**MES (7) vs LES (9)**					UD	UD	UD	UD	UD	UD
**Male (7) vs Female (9)**					A p=0.85, pwr 0.06	KW p=0.01*, g 1.07	KW p=0.5, pwr 0.07	A p=0.5, pwr 0.16	KW p=0.96, pwr 0.23	KW p=0.7, pwr 0.06
**Age (ST 0.007, g 1.6)**					LM p=0.19	LM p=0.1	LM p=0.6	LM p=0.63	LM p=0.4	LM p=0.9
**Ctrl (8) vs Null (8)**					A p=0.38, pwr 0.17	ST p=0.35, pwr 0.14	KW p=0.07, pwr 0.09, g 0.25	A p=0.57, pwr 0.11	KW p=0.2, pwr 0.2	KW p=0.6, pwr 0.12
**II**		**+/+,+/−**	9	7–47 (20)	1.6 ± 0.2	200 ± 50	240 ± 40	17 ± 5	11 ± 3 (7)	700 ± 400 (7)
		**−/−**	8	6–60 (39)	2.5 ± 0.5	290 ±100	240 ± 40	20 ± 5	4 ± 2 (7)	200 ± 100 (7)
**WT (4) vs HET (5)**					ST p=0.3, pwr 0.17	ST p=0.4, pwr 0.19	ST p=0.07, g 1.4, pwr 0.5	ST p=0.7, pwr 0.1	ST p=0.8, pwr 0.06	KW p=0.72, pwr 0.07
**MES (7) vs LES (10)**					A p=0.53, pwr 0.08	A p=0.6, pwr 0.05	KW p=0.85, pwr 0.06	A p=0.12, pwr 0.44	A p=0.64, pwr 0.08	KW p=0.99, pwr 0.08
**Male (6) vs Female (8)**					A p=0.99, pwr 0.08	A p=0.99, pwr 0.1	A p=0.7, pwr 0.13	KW p=0.65, pwr 0.1	A p=0.84, pwr 0.07	KW p=0.72, pwr 0.22
**Age (W 0.28)**					LM p=0.97	LM p=0.58	LM p=0.38	LM p=0.04*, 0.4%/day	LM p=0.31	LM p=0.29
**Ctrl (8) vs Null (8)**					A p=0.053, g 1.15, pwr 0.6	KW p=0.92, pwr 0.1	A p=0.16, pwr 0.13	A p=0.72, pwr 0.06	KW p=0.11, pwr 0.4	KW p=0.48, pwr 0.22

**Table 2.** Step-evoked receptor potentials in extrastriolar hair cells. Mean ± SEM (number of cells, if different from the set). *g*, Hedge’s g affect size; *ST*, Student’s t-test; *A*, ANOVA (Tukey’s HSD); *W*, Welch ANOVA (Games-Howell test); *KW*, Kruskal-Wallis (Dunn’s test); *LM*, linear regression. Factors were not analyzed if there was *ID*, insufficient data, or *UD*, unequal distribution.

				V(X) Boltzmann parameters			
Type	*Kcna10*	n	Age (median)	Max V_RP_ (mV)	X_1/2_ (nm)	S (nm)	τ_onset_ (ms)
**I**	**+/+, +/−**	8	7–28 (18)	8 ± 1	460 ± 50	250 ± 20	0.6 ± 0.3
	**−/−**	8	15–60 (45)	41 ± 5	100 ± 30	270 ± 40	1.8 ± 0.3
**WT (1) vs HET (7)**				ID	ID	ID	ID
**MES (7) vs LES (9)**				UD	UD	UD	UD
**Male (7) vs Female (9)**				KW p=0.03*, g 1	A p=0.7, pwr 0.73	A p=0.12, power 0.3	ST p=0.01*, g 1.5
Age (ST p=0.007**, g 1.6)				LM p=0.34	LM p=0.06	LM p=0.76	LM p=0.71
**Ctrl (8) vs Null (8)**				ST p=6E-6****, g 3.6	A p=6E-5****, g 3	A p=0.73, pwr 0.07	KW p=0.02*, g 1.6
**II**	**+/+,+/−**	8	7–47 (20)	40 ± 6	150 ± 60	290 ± 50	3 ± 2 (4)
	**−/−**	7	6–60 (39)	50 ± 10	130 ± 80	170 ± 30	3 ± 3 (6)
**WT (3) vs HET (5)**				ST p=0.9, pwr 0.06	ST p=0.34, pwr 0.25	ST p=0.21, pwr 0.23	ID
**MES (6) vs LES (9)**				A p=0.85, pwr 0.06	A p=0.22, pwr 0.33	KW p=0.1, pwr 0.3	ST p=0.14, pwr 0.3
**Male (6) vs Female (6)**				A p=0.35, pwr 0.19	A p=0.8, pwr 0.08	KW p=0.26, pwr 0.06	ID
**Age (W p=0.28)**				LM p=0.58	LM p=0.2	LM p=0.16	LM p=0.66
**Ctrl (8) vs Null (8)**				ST p=0.56, pwr 0.16	A p=0.85, pwr 0.07	ST p=0.06, g 1.1, pwr 0.5	KW p=0.7, pwr 0.1

**Table 3.** Frequency tuning of receptor potentials and postsynaptic potentials. V_0_ was measured from the first 200 ms of the frequency sweep. Hypothesis-tests are below the table: *ST*, Student’s t-test (two-tailed unless otherwise stated); *KW*, Kruskal-Wallis ANOVA.

		Gain re: X - Eq. 4			Phase re: X - Eq. 5				
Cell Type	Kcna10	AC gain (mV/um)	*f*_LP_ (Hz)	V0 (mV)	Phase span (deg)	*f*_LP_ (Hz)	Baseline phase (deg)	N	Age range (median)
**I**	+/+,+/−	3.28	150	−89 ± 1	25	4	32	8	P7–28 (18)
	−/−	23.5	25	−45 ± 5	69	10	59	7	P15–60 (45)
**II**	+/+,+/−	30.72	66.97	−63 ± 3	91.9	38	68	9	P17–45 (20)
	−/−	45.7	20.9	−52 ± 4	73.9	20.3	53.7	8	P6–60 (39)
**Calyx**	+/+,+/−	3.64	18	−68 ± 1	36	9.9	6	5	P52–105 (99)
	−/−	3.94	6	−66 ± 2	34	10.6	−5	5	P15–220 (37)

**Type I HCs**. Mixed ANOVA and pairwise Tukey’s HSD test.

*Gain*, Pairwise 2–100 Hz: p = 2E-7****; 5E-8****, 3E-7****, 2E-5****, 8E-4**, 2E-3**, 0.02*, 0.04*. *Phase*, Pairwise 2–100 Hz: p = 0.85, 1, 1, 0.02*, 2E-4***, 3E-5****, 4E-6****, 8E-7****.

*V*_*0*_, ST p=7E-11, g 5.9

**Type II HCs**. Mixed ANOVA and pairwise Tukey’s HSD test.

*Gain*: Overall, by Genotype, p=0.28; by Frequency, p=5E-8; by Genotype-Frequency Interaction, p=0.002. No pairwise comparisons had p<0.05.

*Phase*: Overall, by Genotype, p = 0.009; by Frequency, p = 5E-26; by Genotype-Frequency Interaction, p = 0.3. No pairwise comparisons had p<0.05.

*V*_*0*_, KW p=0.04, g 1.2

**Calyx**: Mixed ANOVA.

*Gain*: Overall, by Genotype, p=0.49; by Frequency, p=3E-22; by Genotype-Frequency Interaction, p=0.67.

*Phase*: Overall, by Genotype, p=0.24; by Frequency, p=4E-18; by Genotype-Frequency Interaction, p=0.99.

*V*_*0*_, ST p=0.46, pwr 0.1.

**Calyx re: type I HC**: t-tests were used instead of ANOVA because summary statistics were being compared.

Gain: Pairwise 2–100 Hz, Welch one-tailed t-test: p=0.007**, 0.005**, 0.02*, 0.03*, 0.051, 0.054, 0.063, 0.065.

*Phase*: Pairwise 2–100 Hz, ST: p=0.008**, 0.007**, 0.3, 0.4, 0.4, 0.7, 0.3, 0.3.

## Abbreviations:

*f*_LP_: lowpass corner frequency
g_K,L_: low-voltage-activated K^+^ conductance in type I HCs
g_A_: A-type (inactivating) K_V_ conductance in type II HCs
g_DR_: delayed rectifier K_V_ conductance in type II HCs
HC: hair cell
