# Supplementary material for: KV1.8 (Kcna10) potassium channels enhance fast, linear signaling in vestibular hair cells and facilitate vestibulomotor reflexes and balance
Source: bioRxiv. 2025 Jan 28:2025.01.28.634388. Preprint. [Version 1] doi: 10.1101/2025.01.28.634388 (PMC11838376; doi:10.1101/2025.01.28.634388)
Supplement: 1 [file NIHPP2025.01.28.634388V1-supplement-1.pdf]

bioRxiv preprint doi: https://doi.org/10.1101/2025.01.28.634388; this version posted January 28, 2025. The copyright holder for this preprint (which was not certified by peer review) is the author/funder, who has granted bioRxiv a license to display the preprint in perpetuity. It is made available under aCC-BY 4.0 International license.

SUPPLEMENTAL FIGURES

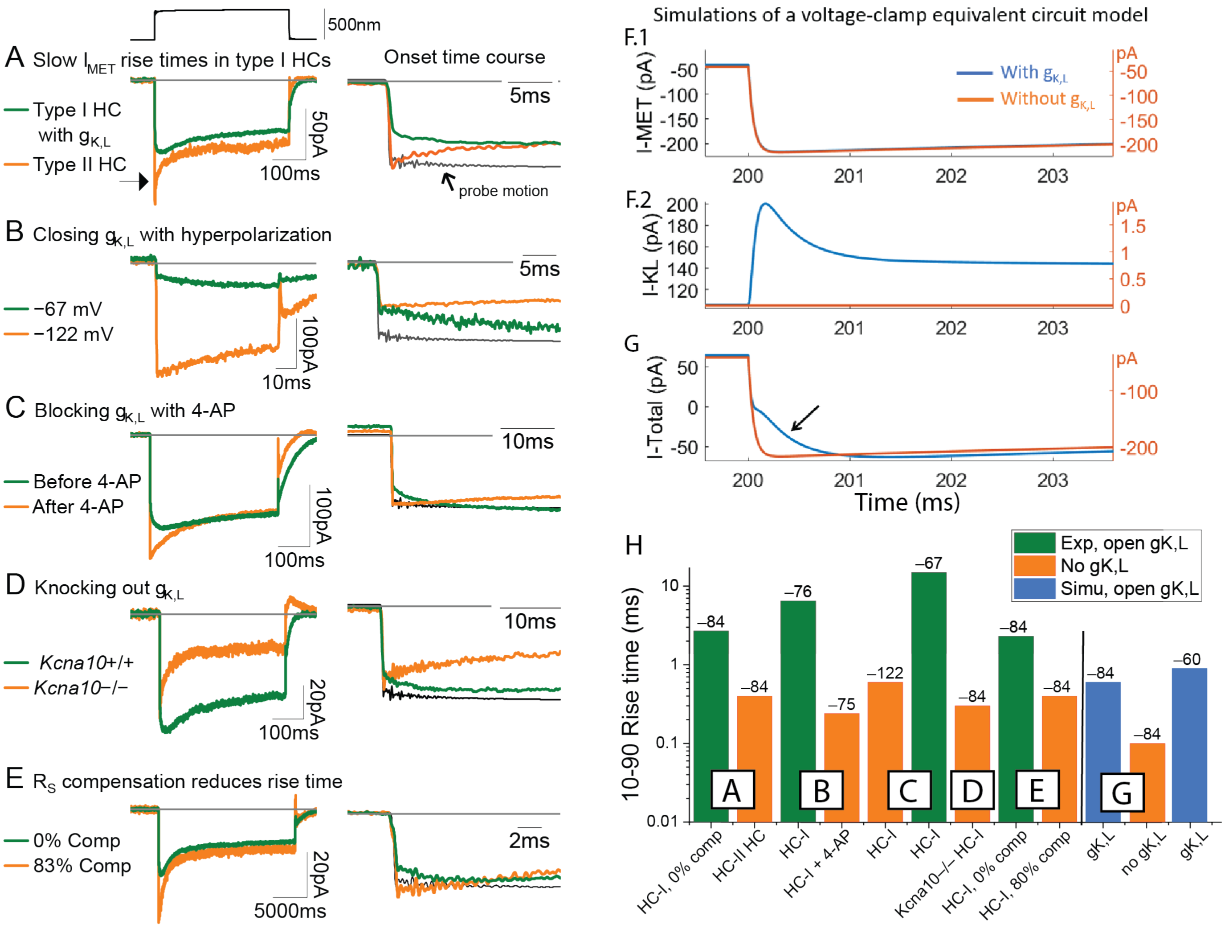

Supplemental Figure 1. $g_{K,L}$ can introduce creep into voltage-clamp recordings of MET currents.

(**A**) MET currents with slow rise times sometimes appeared in type I but not type II hair cells. MET currents had a 10-90% rise time of 2.7 ms in a type I HC and 0.4 ms in a type II HC. Holding potential –84 mV. *Right column*, normalized responses overlaid to show time course at the stimulus onset. (**B**) MET currents' rise times were reduced by closing $g_{K,L}$ channels by hyperpolarizing below their activation threshold. MET current rise times: at –67 mV, 14.9 ms; at –122 mV, 0.6 ms. (**C**) MET currents' rise times were reduced by inhibiting $g_{K,L}$ channels with extracellular 5 mM 4-AP. MET currents' rise times: 6.5 ms before 4-AP; 0.24 ms after 4-AP. Holding potential –74 mV. (**D**) MET currents rise times were smaller in a *Kcna10*$^{-/-}$ type I HC lacking $g_{K,L}$ (0.3 ms) than in a *Kcna10*$^{+/-}$ type I hair cell (2.7 ms). Holding potential –84 mV. (**E**) Series resistance (R_S) compensation corrected slow rise times. A type I HC (P23 LES, 6pF) with R_S = 10 MΩ. MET currents' rise times: at 0% R_S compensation, 2.3 ms; at 80% R_S compensation, 0.4 ms. Holding potential –84 mV. (**F-G**) Simulations of a voltage-clamp equivalent circuit that includes g_{MET}, $g_{K,L}$, R_S, C_m, and time constants of clamp. MET current (**F.1**) and current through $g_{K,L}$ (**F.2**) are summed (**G**) to show current recorded during a whole-cell patch clamp experiment. *Arrow,* creep: the slower, two-phase onset of total current in the presence of $g_{K,L}$. Total current rise times: with $g_{K,L}$, 0.6 ms; without $g_{K,L}$, 0.1 ms. Model parameters in Supplemental Materials. (**H**) 10-90% rise times of experimental and simulated MET current. *Labels above each bar*: holding potential (mV). *Boxed letters*: panel of this figure that these data refer to.

bioRxiv preprint doi: https://doi.org/10.1101/2025.01.28.634388; this version posted January 28, 2025. The copyright holder for this preprint (which was not certified by peer review) is the author/funder, who has granted bioRxiv a license to display the preprint in perpetuity. It is made available under aCC-BY 4.0 International license.

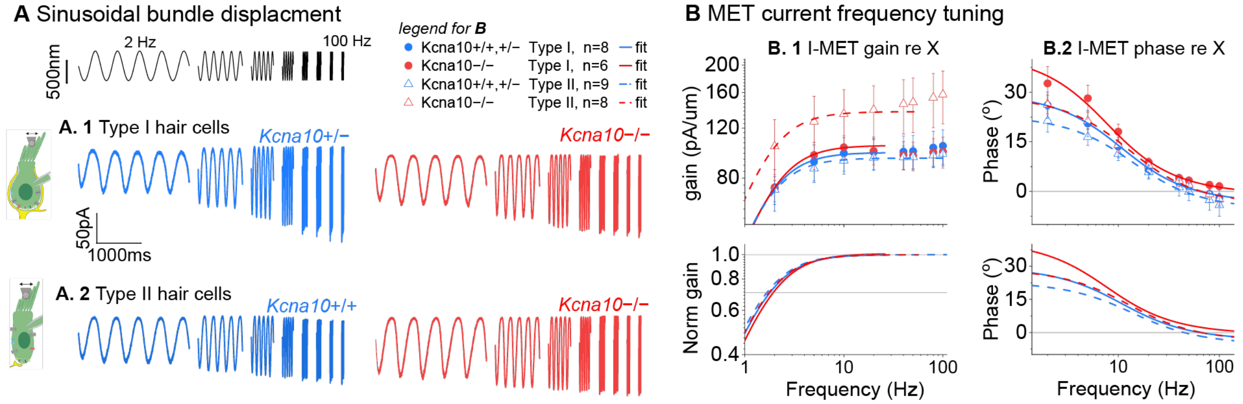

Supplemental Figure 2. *Kcna10* deletion does not affect frequency tuning of MET currents.
(**A**) Exemplar MET currents$_T$ from type I HCs (***A.1***, *left*, P17 LES, V_{hold} −74 mV; *right*, P45 MES, V_{hold} −84 mV) and type II HCs (***A.2***, *left*, P22 MES, V_{hold} −74 mV; *right*, P49 MES, V_{hold} −74 mV). Each trace averages 4-14 presentations. Bundle displacement at top and scale bar in ***A.1*** apply to whole figure. (***B.1***) Gain fit with Eq. 4. (***B.2***) Phase fit with Eq. 5. No effect detected by Genotype, Type, or Zone (3-way mixed ANOVA).

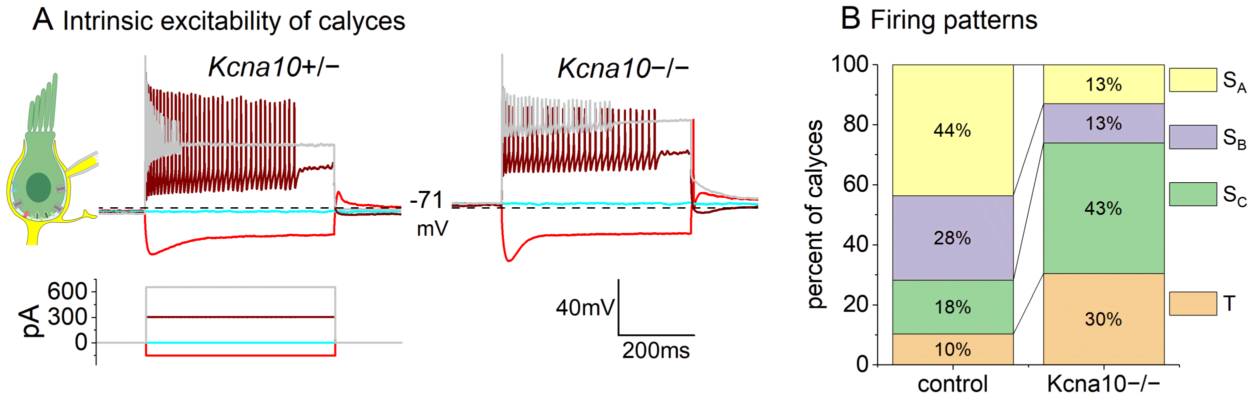

Supplemental Figure 3. *Kcna10* deletion does not affect currents and excitability of calyces.
(**A**) Representative current-clamp records from control and *Kcna10*$^{-/-}$ calyces from the LES. *Bottom panel*, injected current. (**B**) The distribution of firing types (Sustained A, B, C, and Transient) in extrastriolar control (n=39) and *Kcna10*$^{-/-}$ (n=23) calyces. Statistics in Suppl. Table 1.

bioRxiv preprint doi: https://doi.org/10.1101/2025.01.28.634388; this version posted January 28, 2025. The copyright holder for this preprint (which was not certified by peer review) is the author/funder, who has granted bioRxiv a license to display the preprint in perpetuity. It is made available under aCC-BY 4.0 International license.

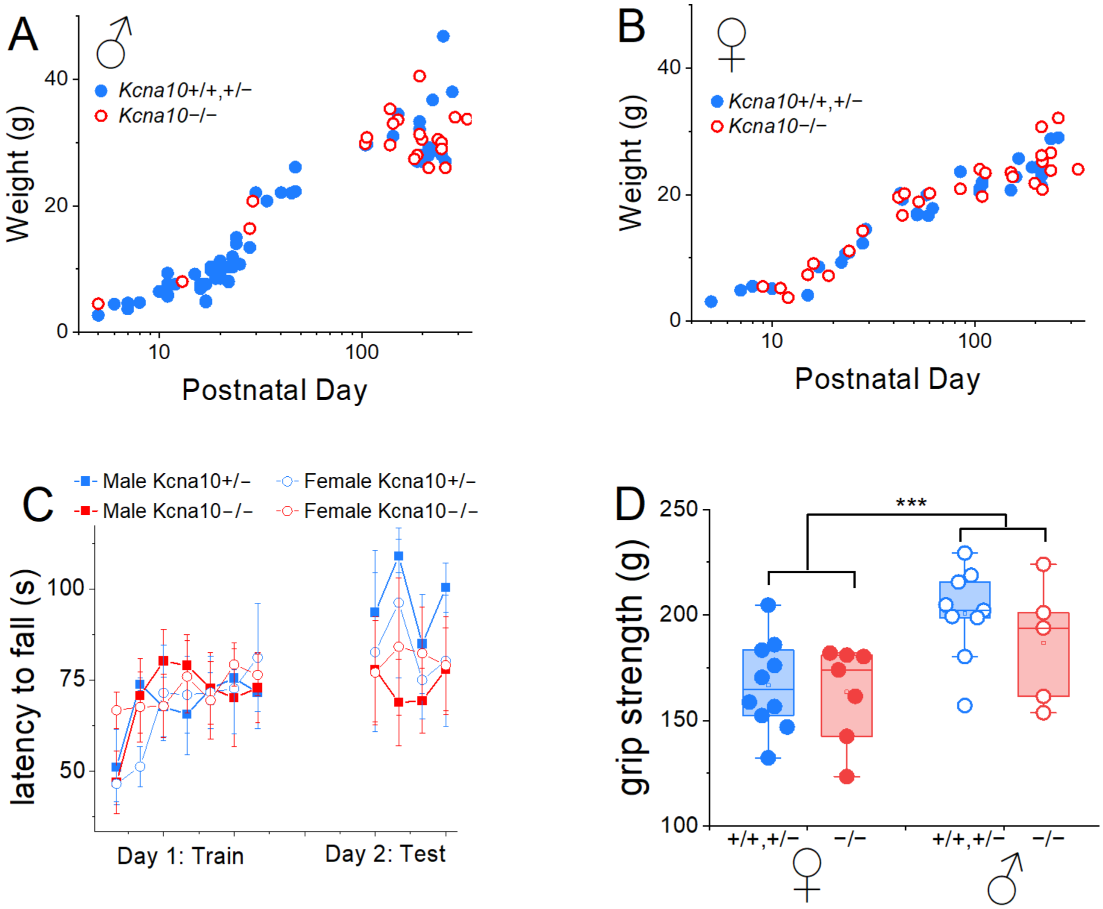

Supplemental Figure 4. *Kcna10*$^{-/-}$ mice have normal weight, motor learning, and strength.

(**A-B**) Weight developed normally in males and females. (**C**) Rotarod learning was similar for *Kcna10*$^{+/-}$ and *Kcna10*$^{-/-}$ mice (mean ± SEM). On test trials, latency to fall: *Kcna10*$^{+/-}$ (80 ± 7 s; 3 males, 7 females; age 2-6.9 months, median 3.8); *Kcna10*$^{-/-}$ (82 ± 9 s; 5 males, 5 females; age 2-6.9 months, median 3.8), ANOVA Tukey's test, p=0.85, power 0.05. (**D**) Grip strength was greater in males (n=14) than females (n=17; 2-way ANOVA, Tukey's p<0.001, g 1.36), but similar between controls (n=19) and nulls (n=12; 2-way ANOVA, Tukey's p=0.36, power 0.16). *Asterisks*, *** P<0.001

bioRxiv preprint doi: https://doi.org/10.1101/2025.01.28.634388; this version posted January 28, 2025. The copyright holder for this preprint (which was not certified by peer review) is the author/funder, who has granted bioRxiv a license to display the preprint in perpetuity. It is made available under aCC-BY 4.0 International license.

Extended Data Tables

Supplemental Table 1. Statistics of voltage-dependent currents and excitability in utricular calyces from the lateral and medial extrastriola. Mean ± SEM (number of cells). *KW*, Kruskal-Wallis ANOVA with posthoc Dunn's test; *ST*, Student's t-test; *ID*, insufficient data; *LM*, linear regression.

Kcna10	Age range (days, median)	V_m (mV)	R_{in} (MΩ)	Rheobase (pA)	Sag Ratio	I_H (pA) at –124 mV	K_V Half (mV)	K_V g_{max} (nS)	C_m (pF)
**+/+,+/–**	8-259 (17)	–69.8 ± 0.7 (39)	290 ± 20 (39)	0.7 ± 0.2 (34)	0.3 ± 0.02 (38)	–490 ± 60 (25)	–40.3 ± 0.3 (22)	13 ± 3 (25)	4 ± 1 (27)
**–/–**	12-259 (38)	–67 ± 1 (21)	240 ± 30 (22)	1.7 ± 0.3 (17)	0.33 ± 0.03 (22)	–620 ± 60 (17)	–38.7 ± 0.8 (12)	14 ± 2 (12)	5 ± 1 (23)
**WT (13) vs Het (26)**		ST p=0.26, pwr 0.22	ST p=0.43, pwr 0.12	KW 0.p=28, pwr 0.53	ST p=0.73, pwr, 0.11	KW p=0.1, pwr 0.3	ST p=0.27, pwr 0.18	KW p=0.89, pwr 0.09	KW p=0.16, pwr 0.26
**MES (5) vs LES (51)**		ST p=0.016, g 1.2	KW p=0.72, pwr 0.04	KW p=0.94, pwr 0.003	ST p=0.99, pwr 0.04	ID	ID	ID	ID
**Male (33) vs Female (17)**		ST p=0.5, pwr 0.1	KW p=0.88, pwr 0.06	ST p=0.09, pwr 0.41	ST p=0.27, pwr 0.22	KW p=0.45, pwr 0.03	ST p=0.3, pwr 0.19	KW p=0.7, pwr 0.29	KW p=0.62, pwr 0.11
**Age (Ctrl vs Null KW 0.001**)**		LM p=0.7	LM p=0.89	LM p=0.89	LM p=0.07	LM p=0.91	LM p=0.65	LM p=0.9	LM p=0.74
**Ctrl vs Null**		ST p=0.07, pwr 0.46	KW p=0.35, pwr 0.17	KW p=0.41, pwr 0.1	ST p=0.37, pwr 0.16	KW p=0.06, pwr 0.44	ST p=0.87, pwr 0.1	KW p=0.25, pwr 0.03	KW p=0.38, pwr 0.09

bioRxiv preprint doi: https://doi.org/10.1101/2025.01.28.634388; this version posted January 28, 2025. The copyright holder for this preprint (which was not certified by peer review) is the author/funder, who has granted bioRxiv a license to display the preprint in perpetuity. It is made available under aCC-BY 4.0 International license.

Supplemental Table 2. VOR group statistics with ANOVA. *Kcna10*$^{+/+}$ and *Kcna10*$^{+/-}$ were combined as controls because no differences were detected between them.

Type	*Kcna10*	N	Age Range (months, median)	Gain Across Frequency	Phase (deg) Across Frequency
VORd	+/+, +/–	14	10-20 m (18)		
	–/–	9	10-21 m (19)		
			**WT (4) vs HET (10)**	p = 0.87	p = 0.91
			**Ctrl (14) vs Null (9)**	p = 0.000197	p = 7.40e-07
VORl	+/+, +/–	12	10-20 m (18)		
	–/–	9	10-20 m (18)		
			**WT (4) vs HET (8)**	p = 0.31	p = 0.990
			**Ctrl (12) vs Null (9)**	p = 1.78e-07	p= 0.0214

Supplemental Table 3. VOR statistical tests at each frequency.

				Gain									Phase (deg)								
Type	*Kcna10*	N	Age (months, median)	0.4 Hz	0.6 Hz	0.8 Hz	1 Hz	2 Hz	5 Hz	10 Hz	15 Hz	20 Hz	0.4 Hz	0.6 Hz	0.8 Hz	1 Hz	2 Hz	5 Hz	10 Hz	15 Hz	20 Hz
VORd	+/+, +/–	14	10-20 (18)																		
	–/–	9	10-21 (19)																		
			**Ctrl (14) vs Null (9) p-values**	8.50E-06	1.50E-05	0.00017	0.00019	0.012	0.035	0.8	0.58	0.3	0.0005	1.40E-05	1.84E-05	2.90E-05	0.04	0.98	0.15	0.41	0.3
VORl	+/+, +/–	12	10-20 (18)																		
	–/–	9	10-20 (18)																		
			**Ctrl (12) vs Null (9) p-values**	0.052	0.035	0.033	0.043	0.036	0.51	0.95	0.47	0.09	0.51	0.16	0.21	0.12	0.022	0.16	0.72	0.45	0.12

bioRxiv preprint doi: https://doi.org/10.1101/2025.01.28.634388; this version posted January 28, 2025. The copyright holder for this preprint (which was not certified by peer review) is the author/funder, who has granted bioRxiv a license to display the preprint in perpetuity. It is made available under aCC-BY 4.0 International license.

Supplemental Table 4. Gait during forced run on a treadmill (25 cm/s) was similar across genotypes, sex, and age. Mean ± SEM. *ST*, Student's t test; *LM*, linear model.

Kcna10	N	Age (median, range)	Paw	Paw angle (degrees)	Paw stance width (mm)	Stride duration (ms)	Swing duration (ms)	Stance duration (ms)
+/+,+/–	16	84, 68-211	*Fore:*	22.2 ± 0.6	7.5 ± 0.2	257 ± 5	97 ± 3	160 ± 5
			Hind:	26 ± 1	13.3 ± 0.2	260 ± 4	62 ± 5	200 ± 5
–/–	12	84, 70-211	*Fore:*	21.7 ± 0.6	7.7 ± 0.2	254 ± 4	89 ± 3	165 ± 5
			Hind:	24 ± 1	12.9 ± 0.3	258 ± 5	57 ± 3	206 ± 5

Supplemental Table 5. **Exploration of an open field arena for 1 hour.** Mean ± SEM (number of mice). *g*, Hedge's g effect size; *ST*, Student's t-test with equal variance; *W*, t-test with Welch correction; *KW*, Kruskal-Wallis ANOVA with postdoc Dunn's Test with Bonferroni correction; *A*, ANOVA with posthoc Tukey's HSD test; *LM*, linear regression model for continuous variables (coefficient provided after the p-value if significant); *pwr*, power.

Kcna10	Rearing event count	Time rearing (min)	Center-phobia index	Distance travelled (m)	Average velocity (cm/s)	N	Age range (month, median)
**+/–**	550 ± 70	2.6 ± 0.5	11 ± 2	42 ± 5	1.15 ± 0.09	4M, 4F	1.4-3.7 (2.1)
**–/–**	330 ± 30	1.3 ± 0.2	9 ± 2	37 ± 6	1.05 ± 0.05	4M, 4F	1.4-3.7 (1.9)
**Male vs Female**	A p=0.27, pwr 0.17	A p=0.02*, g 1.04	A p=0.07, pwr 0.45	A p=0.3, pwr 0.18	A p=0.08, pwr 0.4		
**Age (month)**	LM p=0.7	LM p=0.06	LM p=0.005**, −4	LM p=0.07	LM p=0.09		
**Ctrl vs Null**	A p=0.01*, g 1.4	W p=0.02*, g 1.4	A p=0.49, pwr 0.11	A p=0.57, pwr 0.09	A p=0.3, pwr 0.19		

bioRxiv preprint doi: https://doi.org/10.1101/2025.01.28.634388; this version posted January 28, 2025. The copyright holder for this preprint (which was not certified by peer review) is the author/funder, who has granted bioRxiv a license to display the preprint in perpetuity. It is made available under aCC-BY 4.0 International license.

Supplemental Table 6. Quantitative descriptions of crossing a narrow balance beam in *Kcna10*$^{+/+,+/-}$ and *Kcna10*$^{-/-}$ mice. Mean ± SEM (number of mice); *Median*. g is effect size, Hedge's g. T-test assumed equal variance unless stated as Welch correction. KWA is Kruskal-Wallis ANOVA with postdoc Dunn's Test with Bonferroni correction. The significance of continuous predictors (Age and Weight) were determined by ANOVA comparison of linear models with and without each predictor.

Kcna10	Instability score	Paw Slips	Tail Wraps	Beam Slips	Success Rate	Time To Cross (s)	N	Age range (month, median)
+/+,+/–	1.8 ± 0.3<br>*1.4*	1.0 ± 0.1<br>*0.9*	0.6 ± 0.2<br>*0.3*	0.2 ± 0.1<br>*0*	0.86 ± 0.06<br>*1*	14 ± 2<br>*12*	10M, 14F	2-6.9 (3.8)
–/–	4.3 ± 0.6<br>*3.6*	1.6 ± 0.3<br>*1.3*	1.7 ± 0.3<br>*1*	1 ± 0.4<br>*0.3*	0.56 ± 0.08<br>*0.67*	20 ± 4<br>*14*	8M, 11F	2-6.9 (3.8)
**Male (n=18) vs Female (n=25)**	KW 0.12, pwr 0.33	KW 0.76, pwr 0.09	KW 0.13, pwr 0.24	KW 0.1, pwr 0.55	KW 0.1, pwr 0.34	KW 0.052, g 0.84, pwr 0.74		
**WT (n=4) vs HET (n=20)**	KW 0.39, pwr 0.2	T-test 0.2 pwr 0.15	KW 0.3, pwr 0.17	KW 0.8, pwr 0.01	KW 0.94, pwr 0.05	KW 0.4, pwr 0.1		
**Age (Ctrl vs Null t-test 0.8)**	P 0.16	P 0.5	P 0.5	P 0.12	P 0.0078 **	P 0.069		
**Weight (Ctrl vs Null KW 0.9)**	P 0.015 *	P 0.8	P 0.1	P 0.002 **	P 0.0016 **	P 0.012 *		
**Ctrl (n=24) vs Null (n=19)**	KW 0.00037, g 1.2 ***	KW 0.09, pwr 0.4	KW 0.0007, g 1.0 ***	KW 0.002, g 0.7**	KW 0.002, g 0.9 **	KW 0.096, g 0.5		

Supplemental Table 7. Swim body posture differed between Kcna10+/– and Kcna10–/– mice. Mean (median) ± SEM. *g*, Hedge's g effect size; *ST*, Student's t-test with equal variance; *W*, t-test with Welch correction; *KW*, Kruskal-Wallis ANOVA with postdoc Dunn's Test with Bonferroni correction; *A*, ANOVA with posthoc Tukey's HSD test; *LM*, linear regression model for continuous variables (coefficient provided after the p-value if significant); *pwr*, power.

Kcna10	N	Age range (month, median)	Waterline-Chin-Hindleg angle (°)	Waterline-Chin-Tail angle (°)	Waterline-Neck-Nose angle (°)	Waterline-Neck-Tail (°)
+/+,+/–	8M, 7F	2.9-8.4 (5)	23 (20) ± 2	12 (11) ± 2	165 (166) ± 2	21 (23) ± 3
–/–	5M, 4F	2.9-8.4 (5.4)	31 (30) ± 3	19 (19) ± 3	156 (154) ± 2	30 (32) ± 5
	**WT (n=7) vs HET (n=8)**		KW 0.35, pwr 0.11	KW 0.2, pwr 0.16	KW 0.56, pwr 0.06	ST 0.17, pwr 0.3
	**Male vs Female**		A 0.003**, g 1.14	A 0.004**, g 1.2	A 0.2, pwr 0.22	A 0.0008***, g 1.5
	**Age (Ctrl vs Null KW 0.045)**		LM 0.16	LM 0.34	LM 0.92	LM 0.35
	**Weight (Ctrl vs Null ST 0.8)**		LM 0.02*, 1.2	LM 0.02*, 1.2	LM 0.32	LM 0.009, 1.7
	**Ctrl (15) vs Null (9)**		KW 0.007**, g 1.2	KW 0.02*, g 0.9	KW 0.002**, g 1.6	A 0.03*, g 0.82

bioRxiv preprint doi: https://doi.org/10.1101/2025.01.28.634388; this version posted January 28, 2025. The copyright holder for this preprint (which was not certified by peer review) is the author/funder, who has granted bioRxiv a license to display the preprint in perpetuity. It is made available under aCC-BY 4.0 International license.

Supplemental Table 8. Model parameters for the voltage-equivalent circuit used for simulations of current through MET and $g_{K,L}$ channels in Suppl. Fig. 1. Model adapted from [63] .

Symbol	Value	Description
**Capacitance**		
C_f	0.3 pF	Feedback shunt capacitance[63]
C_m	5 pF	Membrane capacitance
C_p	3-5 pF	
**Current**		
I_{in}		Voltage-clamp current
I_{ion}		Whole-cell ion channel current
I_{leak}		Leakage current through imperfect seal
I_m		Membrane current
I_{out}		Recorded current
**Resistance**		
R_f	500 MΩ	Feedback resistance (current gain on HEKA EPC 10)
R_S	10 MΩ	Series resistance between pipette electrode and cell
α	0-0.8	Fraction of R_S compensation
R_{seal}	0.5-10 GΩ	Seal resistance of the pipette tip
**Time constant**		
τ_a	50 µs	Membrane access time constant, $\tau_a = R_s C_m$
τ_{sum}	40 µs	Response time of the summing amplifier[63]
τ_{clamp}	10 µs	Voltage-clamp time constant, $\tau_{clamp} = (1 - \alpha)* \tau_a$
τ_z	150 µs	Transconductor time constant, $\tau_z = R_f C_f$
**Voltage**		
V_{cmd}		Command voltage, follows the voltage-clamp protocol
V_{clamp}		Clamp voltage
V_m		Membrane potential
V_{off}		Offset voltage: amplifier offsets, electrode offsets, junction potentials
V_{out}		Recorded voltage, $V_{out} = I_{out} R_f$
V_p		Pipette potential
**Conductance: $g_{K,L}$**		
g_{max}	300 nS	Maximal conductance [23]
V_{half}	–84 mV	Midpoint voltage activation
dV	5 mV	Slope factor of voltage-dependence
τ_{act}	300 ms	Time constant of activation
E_K	–86 mV	K^+ reversal potential
**Conductance: g_{MET}**		
g_{max}	3 nS	Maximal conductance
$X_{1/2}$	0.3 µm	Midpoint displacement activation
dX	0.2 µm	Slope factor of displacement sensitivity
τ_{Act}	50 µs	Activation time constant
τ_{Adapt}	10 ms	Adaptation time constant
E_{met}	2.6 mV	I_{MET} reversal potential [52]
**Leak through seal**		
E_{Leak}		V_m
g_{Leak}	2 nS	$1/R_{Seal}$, [63]

bioRxiv preprint doi: https://doi.org/10.1101/2025.01.28.634388; this version posted January 28, 2025. The copyright holder for this preprint (which was not certified by peer review) is the author/funder, who has granted bioRxiv a license to display the preprint in perpetuity. It is made available under aCC-BY 4.0 International license.
